# Harnessing big data for enhanced genome-wide prediction in winter wheat breeding

**DOI:** 10.1007/s00122-025-05007-6

**Published:** 2025-08-22

**Authors:** Ravindra Reddy Gundala, Ulrike Avenhaus, Jost Doernte, Wera Maria Eckhoff, Jutta Foerster, Mario Gils, Michael Koch, Martin Kirchhoff, Sonja Kollers, Nina Pfeiffer, Matthias Rapp, Monika Spiller, Valentin Wimmer, Markus Wolf, Yusheng Zhao, Jochen Christoph Reif

**Affiliations:** 1 Leibniz Institute for Plant Genetics and Crop Plant Research, Corrensstraße 3, 06466 Seeland, Germany; 2 W. Von Borries-Eckendorf GmbH & Co. KG, Hovedisser Str. 94, 33818 Leopoldshoehe, Germany; 3 Deutsche Saatveredelung AG, Weissenburger Straße 5, 59557 Lippstadt, Germany; 4 KWS Saat SE, Grimsehlstr. 31, 57574 Einbeck, Germany; 5 SU BIOTEC GmbH, Am Schwabeplan 6B, 60439 Gatersleben, Germany; 6 Nordsaat Saatzucht GmbH, Böhnshauser Str. 1, 38895 Langenstein, Germany; 7 KWS Lochow GmbH, Wetze 3, 37154 Northeim, Germany; 8 Present Address: Nordzucker AG, Magdeburger Landstraße 1-5, 39164 Wanzleben-Boerde, Germany; 9 Present Address: Aardevo B.V. Johannes, Postweg 8, 8308 PB Nagele, Netherlands

## Abstract

**Key message:**

By combining data from different public and private breeding programs for genomic selection, we have increased the size and diversity of the training population, which has led to better predictions of grain yield and plant height in winter wheat compared to using individual training sets.

**Abstract:**

The accuracy of genome-wide prediction is anticipated to improve with an increase in training population size. In our study, we assembled a comprehensive wheat data set consisting of about 18,000 inbred lines and phenotypic data from about 250,000 plots. We evaluated the potential to train genome-wide prediction models using this big data set through data from post-registration trials conducted across a wide range of environments. Our findings demonstrated that using big data can enhance the prediction ability by up to 97% for grain yield and 44% for plant height, outperforming individual training sets. This improvement is primarily attributed to the expansion of the training set size relative to the genetic diversity. In conclusion, big data holds significant potential to accelerate genetic gain in winter wheat predictive breeding, making it a compelling option.

**Supplementary Information:**

The online version contains supplementary material available at 10.1007/s00122-025-05007-6.

## Introduction

Wheat is a global staple food, and to meet the demands of a growing population by 2050, wheat production must be doubled. Achieving this goal at the current rate of yield increase represents a significant challenge (Ray et al. 2013). Research suggests that genomic selection could potentially triple the genetic gain compared to existing phenotypic selection (Meuwissen et al. 2001; Voss-Fels et al. 2019; Tessema et al. 2020). Therefore, it is proposed as a promising strategy to help bridge the yield gap. However, the primary challenge lies in enhancing the prediction ability of genomic selection models to maximize the genetic gain.

Prediction ability depends on a variety of factors and their interactions, including heritability, the genetic architecture of the trait, marker density, population structure, genetic relationship between training and test sets, size of the training population, and genetic diversity (Combs and Bernardo 2013; Lian et al. 2014; Windhausen et al. 2012; Würschum et al. 2017; Werner et al. 2020; Zhao et al. 2021; Alemu et al. 2024). Prediction ability can be enhanced by increasing the training set size in proportion to the genetic diversity (Zhao et al. 2021). This can be achieved cost-effectively by combining phenotypic and genotypic data from breeding programs and public–private partnerships into big data, as demonstrated by Zhao et al. (2021) and Lell et al. (2025). However, in these studies, utilization of test sets drawn from the accumulated data complicates the ability to determine the extent to which a comprehensive training set including a substantial number of early-stage genotypes from breeding programs can accurately predict the true genetic values of those genotypes, especially when accounting for genotype-by-environment interactions. Further, the improvement in the prediction ability appears to be saturated well before reaching the expected maximum defined by the square root of the heritability (Lell et al. 2025). One possible explanation for this observation is the moderate heritability of the test datasets used, which typically is based on a limited number of environments. Furthermore, these imprecise test datasets also hamper efforts to identify additional factors that could enhance prediction ability. These reasons underscore the need for using test datasets where diverse genotypes are tested in a wide range of environments.

In addition to the need for appropriate test datasets, the impact of merging heterogeneous SNP array platforms, which results in a block-like missing value structure (Lell et al. 2025), remains understudied. The integration of disparate genotypic data sets introduces a significant number of non-random missing values and minor alleles, ultimately compromising the overall quality of the genotypic data. This limitation hinders the potential for achieving optimal prediction abilities. Therefore, there is a need for a robust strategy to filter out missing values and minor alleles.

Moreover, several studies have shown that the inclusion of genome-wide epistasis can improve the prediction ability, and this magnitude of improvement can range from 4 to 25%, depending on the crop, population and trait under consideration (Crossa et al. 2010; Jiang and Reif 2015; Raffo et al. 2022). Whereas other studies demonstrated that genome-wide epistasis can be detrimental to the prediction ability (Lorenzana and Bernardo 2009). Further, Santantonio et al. (2019) and Cuevas et al. (2024) modelled epistasis within the subgenome level and found no improvement compared to modelling genome-wide epistasis. However, in most of these studies, the conclusions are drawn based on a single population, but the impact of epistasis on genomic predictions at the scale of big data, particularly when integrating diverse populations, remains unexplored.

In this study, the existing wheat big data set was expanded (Zhao et al. 2021; Lell et al. 2024), resulting in data from approximately 18,000 wheat lines evaluated in approximately 250,000 yield plots. The objective was to combine it with a very powerful test population from post-regional trials to assess the potential of big data. Specifically, our objectives were to: (1) develop a strategy for filtering missing values and minor alleles resulting from the merging of unbalanced genotypic data from disparate sources to reach optimum prediction ability; (2) identify the key drivers of prediction ability in genomic predictions; and (3) examine the value of incorporating genome-wide, subgenome, and chromosome-level epistasis, in addition to additive effects, in genomic predictions at the level of big data.

## Materials and methods

### Plant materials

The genotypic and phenotypic data from which the training populations were assembled came from eight large-scale experimental series of winter wheat conducted in Central Europe (Exp-1 to 8). The genotypic and phenotypic data of the first four series (Exp-1 to 4) have been described in detail previously (Zhao et al. 2015, 2021; Gogna et al. 2022). Exp-5 to 8 comprise genotypic and phenotypic data from four wheat breeding programs generated in 2020 and 2021 (Lell et al. 2025). The data has been extended by ~ 9000 lines phenotyped in ~ 50,000 plots in 2022 and 2023 as described in detail below.

Exp-5 consists of 3211 winter wheat lines that have been tested by KWS LOCHOW GmbH (Bergen, Germany) for grain yield and plant height at 5 to 9 locations in Germany and Poland from 2020 to 2023. Each year, the breeding company performed nine trials with 56 to 154 genotypes per trial and one to two replications. The trials were connected by 10 to 11 registered lines. Plot size ranged between 5 to 15 m^2^.

Exp-6 comprises 3906 winter wheat lines evaluated by Deutsche Saatveredelung AG (Lippstadt, Germany) for grain yield and plant height at up to 20 locations in Germany from 2020 to 2023. Each year, the breeding company performed 12 to 17 trials with 25 to 188 genotypes per trial and one to two replications. The trials were connected by up to 5 released lines. Plot size ranged between 5.25 to 18 m^2^.

Exp-7 is based on 2918 winter wheat lines tested for grain yield and plant height in the Nordsaat Saatzucht GmbH (Langenstein, Germany) breeding program from 2020 to 2023. The lines were evaluated in 4 to 17 trials at up to 11 locations in Germany. The trials were connected with 6 to 18 released varieties. Between 50 to 281 lines were tested per trial with one to two replicates. Plot size ranged from 3.75 to 14.3 m^2^.

Exp-8 is based on 8394 winter wheat lines, which have been evaluated by W. von Borries-Eckendorf GmbH & Co. KG (Leopoldshöhe, Germany) for grain yield and plant height at up to 12 sites in 4 to 23 trials from 2020 to 2023. Trials were connected with up to 7 released varieties. Per trial, 49 to 1115 lines were tested in one or two replications. Plot size ranged from 5.8 to 12 m^2^.

As a test set, we utilized phenotypic data from the years 2017 to 2022 obtained from the German post-registration winter wheat trials (Exp-PRT; Supplementary Table 1; Bundessortenversuch 2017), along with the corresponding genotypic profiles. The Exp-PRT data includes grain yield trials across Germany at 26 to 31 locations (Supplementary Fig. 1). In total, the dataset represents approximately 170 environments, where each environment is defined as a unique combination of year and location. Around 20 genotypes were tested each year with intensive and extensive treatments. In our study, we used only data from the intensive treatment, which included treatments with fungicides and growth regulators, depending on local conditions. The integrated Exp-PRT data set consists of 98 genotypes: 90 inbred lines and 8 hybrids. Genotypic and phenotypic data was available for 83 of the 90 inbred lines. The EXP-PRT dataset becomes highly topical and commercially relevant for the industry due to the genotypes tested in vast number of environments across Germany and representing majority of the current German seed propagation area.

### Phenotypic data curation and analysis

We implemented an un-weighted two-stage analysis of the phenotypic data. This decision is based on previous findings showing that the difference between weighted versus unweighted approaches was negligible (Möhring and Piepho 2009). For Exp-1–4 and Exp-PRT, curated data was used (Gogna et al. 2022; Zhao et al. 2021), and within-environment Best Linear Unbiased Estimates (BLUEs) were available. Given the heterogeneity of the experimental designs in Exp-5–8, linear mixed models tailored to each experimental design derived from model (1) were used to calculate BLUEs for each environment for both traits, grain yield and plant height:

1 $${y}_{ijkl} \sim \mu + {g}_{i} + {t}_{j} +{r}_{jk} + {b}_{jkl} + {\varepsilon }_{ijkl},$$

where $y$ *is the plot level trait measurement,* $\mu$ is the mean, ${g}_{i}$ is the effect of *i*th genotype, ${t}_{j}$ is the *j*th trial effect, ${r}_{jk}$ refers to the effect of *k*th replication in *j*th trial, ${b}_{jkl}$ indicates the effect of *l*th block in the *k*th replication in the *j*th trial and $\varepsilon$ relates to the residuals. Further, all the effects except for the *genotype* are fitted random. Outlier correction was performed using method 4 “Bonferroni-Holm with re-scaled median absolute deviation standardized residuals” as described by Bernal-Vasquez, Utz, and Piepho (2016).

Based on intra-environmental BLUEs, across-environmental Best Linear Unbiased Predictions (BLUPs) were computed for each experimental series, as well as for all the combined datasets using the following model (2):

2 $${y}_{ij} \sim \mu + {g}_{i} + {e}_{j} + {\varepsilon }_{ij},$$

In model (2), $y$ *are the* intra-environmental BLUEs*,* $\mu$ is the mean, $g$ are the genotype effects, $e$ are the environment (field × location × year) effects, and $\varepsilon$ relates to the residuals. Further, all the effects are fitted as random. The estimated variance components were used to calculate broad-sense heritability using the following model (3):

3 $${H}^{2}={\sigma }_{g}^{2}/\left({\sigma }_{g}^{2}+\frac{{\sigma }_{\varepsilon }^{2}}{{N}_{Env}}\right),$$

In model (3), ${\sigma }_{g}^{2}$ being the genotypic variance, ${\sigma }_{\varepsilon }^{2}$ refers to the residual variance, and ${N}_{Env}$ the average number of environments a genotype was measured in. This calculated broad-sense heritability (H^2^) does not correspond to the fundamental heritability defined in quantitative genetics. Please note that, due to potential deviations from underlying assumptions such as random mating and Hardy–Weinberg equilibrium, Bernardo (2020) suggested using the term “reliability” instead of “broad-sense heritability” for greater clarity. Of course, the concept of “reliability” as defined by Bernardo (2020) should not be confused with the more commonly used interpretation of “reliability,” which refers to the bias in predicting an individual’s genetic value based solely on genomic data.

All the calculations were made using the R Statistical Software (v4.0.1; R Core Team 2021) and the “AsReml” package (v4.1.0; David Butler 2019).

### Genomic data integration and analysis

The genotypic data was generated using SNP arrays of different densities ranging from 7000 to 90,000 markers derived from the publicly available 90k SNP array (Wang et al. 2014). After merging the SNP array data across all experimental series, the resulting dataset contained 84,754 SNP markers for 28,606 genotypes. Markers without SNP calls, lacking variation, unmapped markers, and those with conflicting positional information were excluded. Genotypes without phenotypic measurements were excluded (Supplementary Table 1). After these filters, a refined set of 30,175 SNP markers for 18,211 genotypes remained. Markers with less than 5% minor allele frequency (MAF) and more than 80% missing values were also excluded, resulting in SNP profiles for 13,105 markers and 18,211 genotypes. Missing values were imputed using the Beagle software (v5.0, Browning et al. 2018). The Rogers’ distance (Rogers 1972) was calculated among all genotypes and a principal coordinate analysis (PCoA) (Gower 1966) was performed using the “cmdscale” function of the R package “stats” (R Core Team 2021). The effective population size ${N}_{e}$ (Hill 1981; Waples 2006) for different experimental series, and the integrated dataset were calculated.

### Genome-wide prediction models

We used an extended genomic best linear unbiased prediction model that includes additive and epistasis effects (A + E model; 4) (Jiang & Reif 2015). In addition, we tested a model that considers only additive effects (A model). The full model is as follows (4):

4 $$y=\upmu +{g}_{A}+{g}_{E}+\varepsilon ,$$

where $y$ is the vector of across-environment genotype BLUPs, $\mu$ is the intercept, ${g}_{A}$ is the vector of additive genetic values, ${g}_{E}$ is the vector of $additive \times additive$ epistatic genetic values, and $\varepsilon$ is the vector of residuals. The random vector ${g}_{A}=({g}_{A1}, {g}_{A2},\dots ,{g}_{An})$ follows a normal distribution ${g}_{A}\sim N(0,{G}_{A}{\sigma }_{A}^{2})$; ${\sigma }_{A}^{2}$ is the additive genetic variance. The covariance matrix ${G}_{A}$ of additive genetic effects was calculated according to VanRaden (2008; “first method”). The random vector ${g}_{E}=({g}_{E1}, {g}_{E2},\dots ,{g}_{En})$ follows a normal distribution ${g}_{E}\sim N(0,{G}_{E}{\sigma }_{E}^{2})$; ${\sigma }_{E}^{2}$ is the $additive \times additive$ epistatic genetic variance. The covariance matrix ${G}_{E}$ of epistatic genetic effects was calculated as the Hadamard product of the additive relationship matrix (${G}_{A}$) as suggested by Henderson (1985) and further proved by Jiang and Reif (2015).

We investigated the relevance of considering epistasis in genome-wide predictions in more detail and divided it into subgenomic (5-8) and chromosomal levels as follows (9-12):

5 $$y=\mu +{g}_{A}+{g}_{E{A}_{G}}+\varepsilon ,$$

6 $$y=\mu +{g}_{A}+{g}_{E{B}_{G}}+\varepsilon ,$$

7 $$y=\mu +{g}_{A}+{g}_{E{D}_{G}}+\varepsilon ,$$

8 $$y=\mu +{g}_{A}+{g}_{E{A}_{G}}+{g}_{E{B}_{G}}+{g}_{E{D}_{G}}+\varepsilon ,$$

Here, $y$ is the vector of across-environment genotype BLUPs, $\mu$ is the intercept, ${g}_{A}$ is the vector of additive genetic values and ${g}_{E{A}_{G}}$, ${g}_{E{B}_{G}}$, and ${g}_{E{D}_{G}}$ are the vectors of $additive \times additive$ epistatic genetic values of subgenome A, B, and D, and $\varepsilon$ is the vector of residuals. The random vectors ${g}_{A}$, ${g}_{E{A}_{G}}$, ${g}_{E{B}_{G}}$, and ${g}_{E{D}_{G}}$ are assumed to follow a normal distribution ${g}_{A}\sim N(0,{G}_{A}{\sigma }_{A}^{2})$, ${g}_{E{A}_{G}}\sim N(0,{G}_{E{A}_{G}}{\sigma }_{E{A}_{G}}^{2})$, ${g}_{E{B}_{G}}\sim N(0,{G}_{E{B}_{G}}{\sigma }_{E{B}_{G}}^{2})$, and ${g}_{E{D}_{G}}\sim N(0,{G}_{E{D}_{G}}{\sigma }_{E{D}_{G}}^{2})$; where ${\sigma }_{A}^{2}$ is the additive genetic variance and ${\sigma }_{E{A}_{G}}^{2}$, ${\sigma }_{E{B}_{G}}^{2}$, and ${\sigma }_{E{D}_{G}}^{2}$ are the $additive \times additive$ epistatic genetic variance of subgenomes A, B, and D, respectively. The related covariance matrices for subgenomes A, B, and D were calculated the same as in model (4), but using only the SNP markers of the respective subgenome. As a next step, we considered epistasis separately for each chromosome within each subgenome as follows (9-12):

9 $$y=1\mu +{g}_{A}+\sum_{i=1}^{7}{g}_{E{A}_{i}}+\varepsilon ,$$

10 $$y=1\mu +{g}_{A}+\sum_{i=1}^{7}{g}_{E{B}_{i}}+\varepsilon ,$$

11 $$y=1\mu +{g}_{A}+\sum_{i=1}^{7}{g}_{E{D}_{i}}+\varepsilon ,$$

12 $$y=1\mu +{g}_{A}+\sum_{i=1}^{7}{g}_{E{A}_{i}}+\sum_{i=1}^{7}{g}_{E{B}_{i}}+\sum_{i=1}^{7}{g}_{E{D}_{i}}+\varepsilon ,$$

Here, $y$ is the vector of across-environment genotype BLUPs, $\mu$ is the intercept, ${g}_{A}$ is the vector of additive genetic values, and ${g}_{E{A}_{i}}$, ${g}_{E{B}_{i}}, {g}_{E{D}_{i}}$, $i=1-7$ are the vectors of $additive \times additive$ epistatic genetic values of the ${i}^{th}$ chromosome of the subgenomes A, B, and D. The random vectors ${g}_{A}$, ${g}_{E{A}_{i}}$, ${g}_{E{B}_{i}}, and {g}_{E{D}_{i}}$ are assumed to follow a normal distribution ${g}_{A}\sim N\left(0,{G}_{A}{\sigma }_{A}^{2}\right), {g}_{E{A}_{i}}\sim N\left(0,{G}_{E{A}_{i}}{\sigma }_{E{A}_{i}}^{2}\right), {g}_{E{B}_{i}}\sim N\left(0,{G}_{E{B}_{i}}{\sigma }_{E{B}_{i}}^{2}\right)$, and ${g}_{E{D}_{i}}\sim N(0,{G}_{E{D}_{i}}{\sigma }_{E{D}_{i}}^{2})$. The related covariance matrices were calculated the same as in model (4), but using only the SNP markers of the *i*th chromosome of the subgenomes A, B, and D, respectively.

### Scenarios for validating genome-wide predictions

In scenario 1, the A + E model (4) was used for the genomic predictions. The data of individual experimental series and combinations of them (Supplementary Table 2) were used as training sets to predict the performance of the Exp-PRT dataset. The Exp-PRT data was particularly useful as a test set because of the large number of environments used to evaluate grain yield. The prediction ability was computed as the correlation between the observed and predicted phenotypic values. For all the training sets the parameters like genotypic variance (σ^2^_g_), broad-sense heritability (H^2^), the average number of test environments per genotype (N_env_), coefficient of variation of BLUPs (CV), population size (N), effective population size (N_e_), and the ratio N/N_e_ were calculated. These parameters were then correlated with the prediction ability to identify associations between the parameters and the prediction ability.

Scenario 2 was used exclusively to study the relevance of epistasis in the genome-wide predictions. We randomly sampled training sets of 500, 1,000, 2,500, 5,000, and 10,000 genotypes 100 times, used genomic prediction models 4–12, and validated the prediction accuracies based on the Exp-PRT dataset. All genotypes occurring in the Exp-PRT data set were excluded from the training data sets of both scenarios. All computations were executed using the R package “BGLR” (Pérez and de los Campos 2014).

## Results

### Absence of genetically distinct subpopulations

**Fig. 1:**
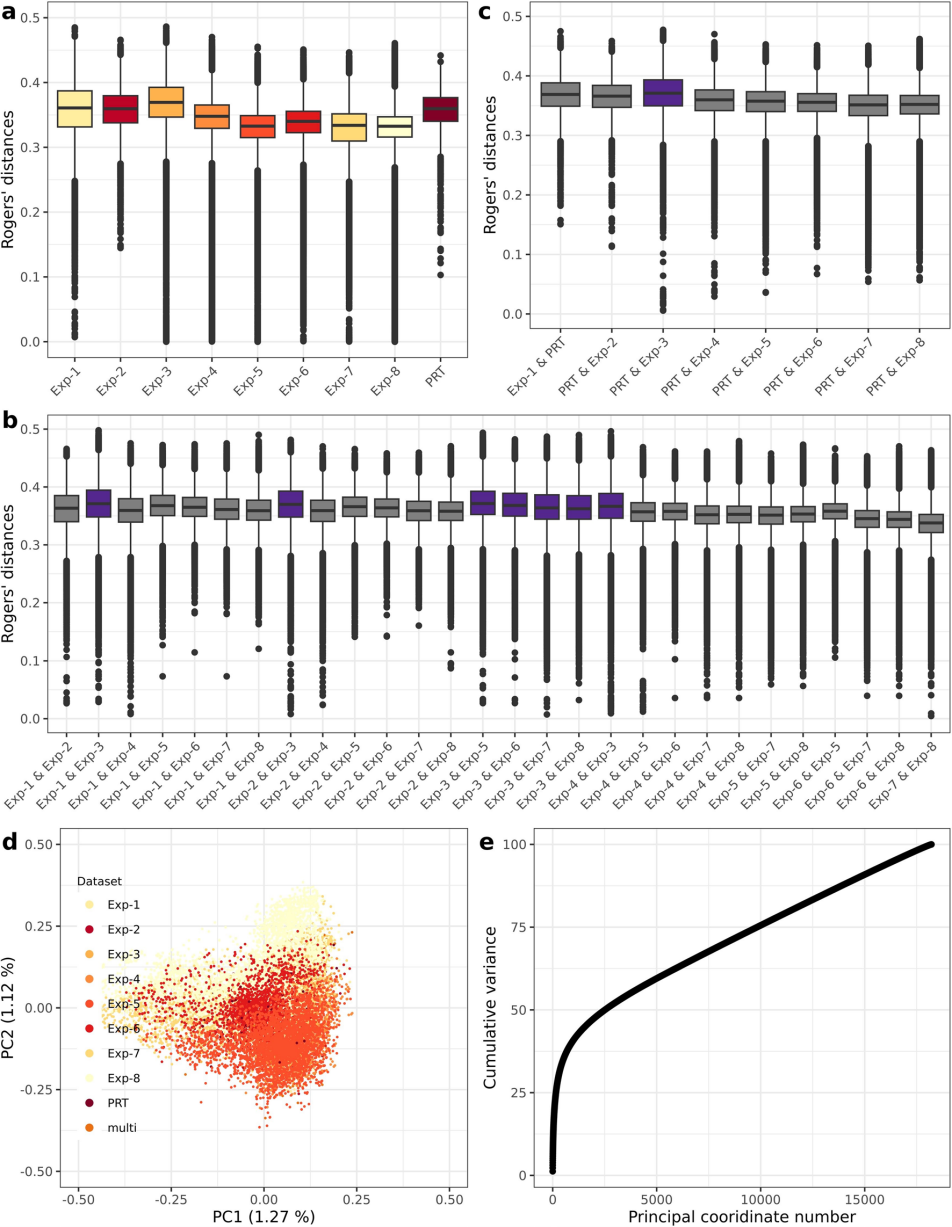
Genetic diversity within and among experimental series (Exp). Distribution of Rogers’ distances **a** within Exp, **b** pairwise between all the other Exp except Exp-PRT, **c** pairwise between Exp-PRT and other Exp. The purple color boxes indicate all pairs involving the Exp-3. **d** Principal coordinate analysis (PCoA) based on the Rogers’ distance matrix of all the inbred lines. The percentage in the parenthesis refers to the proportion of genetic variance explained by the respective principal coordinate (PC). **e** Cumulative variance explained by the PCs

The 18,210 genotypes included in our study cover a wide diversity of winter wheat with an effective population size of *N*_*e*_ = 91 individuals estimated from the SNP data (Supplementary Table 3). All datasets, except Exp-3, contain candidate elite varieties or released varieties developed by different breeding companies for Central Europe. In addition to elite varieties, Exp-3 also includes 239 plant genetic resources maintained at the IPK genebank (Gatersleben, Germany). Accordingly, the distribution of the Rogers’ distances within each experimental series showed a significantly (*p* < 0.05) higher diversity for Exp-3 with an average distance of 0.37 (Exp-3) compared to the other datasets with average distances of 0.33 to 0.36 (Supplementary Table 4, Fig. 1a). Further Exp-PRT showed a significantly (*p* < 0.05) higher diversity than the Exp-5 to 8 (Supplementary Table 4, Fig. 1a). In addition, the average pairwise Rogers’ distances between the genotypes of the different experimental series were significantly higher (*p* < 0.05) at > 0.36 for the comparisons with Exp-3 compared to most of the other pairwise comparisons (Fig. 1b, Supplementary Table 5). The released varieties tested in the Exp-PRT trials showed similar distributions of Rogers’ distances with genotypes of different experimental series (Fig. 1c), with a mean distance ranging from 0.35 to 0.37 (Supplementary Table 6), accompanied by an absence of distinct subpopulations within elite lines (Fig. 1d). Overall, therefore, the use of the Exp-PRT trials as a test set should not bias the results from a genetic point of view and favor a particular composition of training sets.

### Exp-PRT is particularly well suited for use as a test set

**Fig. 2:**
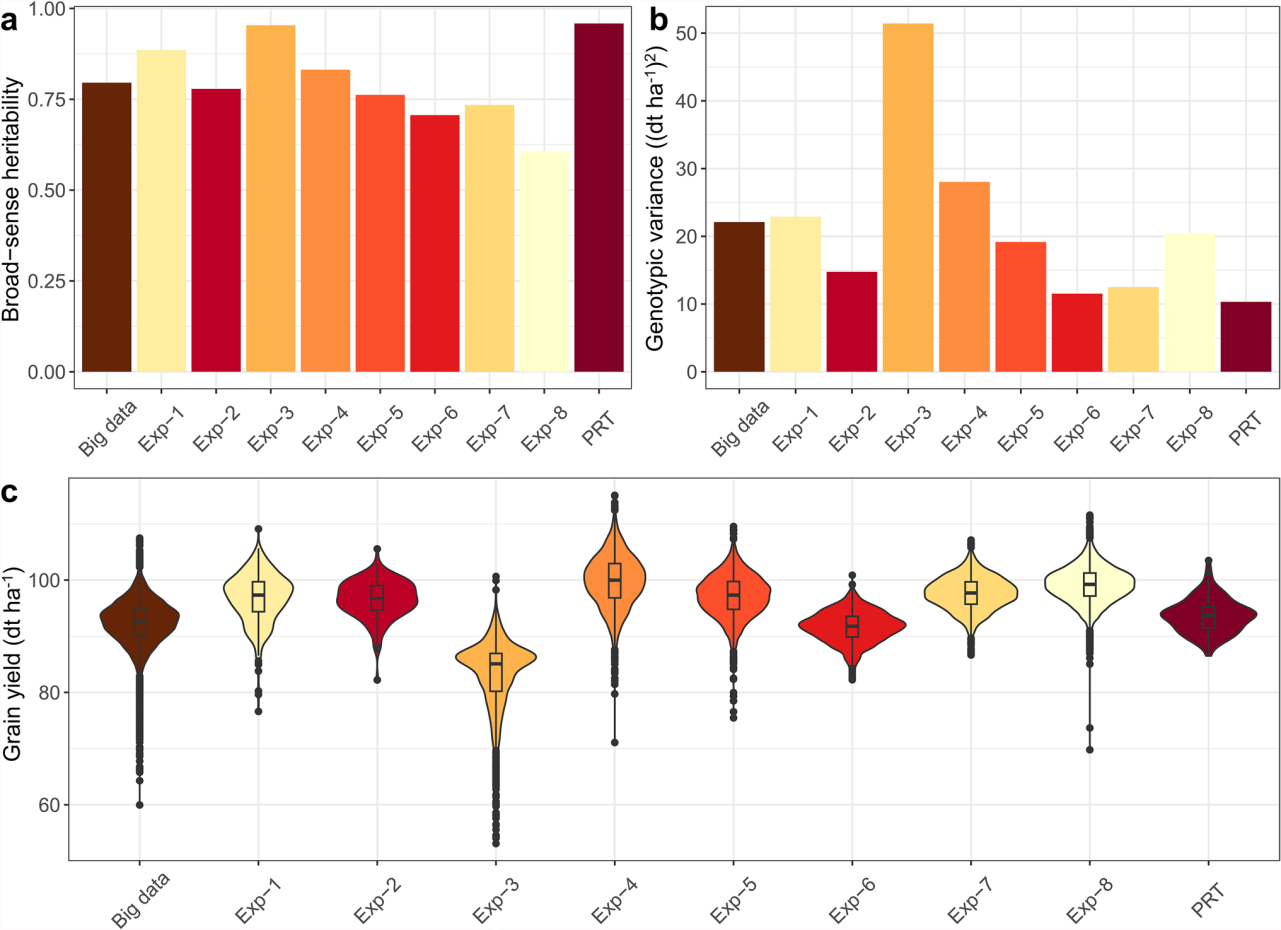
Phenotypic diversity within and across experimental series (Exp). **a** Broad-sense heritabilities, **b** genotypic variance ((dt ha^−1^)^2^), and **c** distribution of best linear unbiased predictions of grain yield (dt ha^−1^) within and across Exp

We assembled extensive grain yield and plant height data from ~ 256,000 plots. Individual genotypes were evaluated for grain yield and plant height on average in 4.8 and 4.5 environments across Central Europe, ranging from ~ 2 (Exp-8) to ~ 39 (Exp-PRT) environments (Supplementary Table 7). The estimated broad-sense heritability within the experimental series ranged from 0.61 (Exp-8) to 0.96 (Exp-PRT) for grain yield and 0.76 (Exp-6) to 0.99 (Exp-1) for plant height. For the analyses across the experimental series, the heritability was 0.80 for grain yield and 0.92 for plant height (Supplementary Fig. 2a, Supplementary Table 7). The genotypic variances within the experimental series for grain yield and plant height were comparable across the series (Fig. 2b-c, Supplementary Fig. 2b-c, Supplementary Table 7), with one outlier from Exp-3 for grain yield and one outlier from Exp-1 for plant height. The pronounced genotypic variation, coupled with high to very high heritabilities for both traits, reflect the excellent quality of the phenotypic data underlying our study. In particular, the high heritabilities for the Exp-PRT dataset, with estimates of 0.96 for grain yield and 0.98 for plant height, make this an ideal test set.

### Merging data across experimental series increases the ability to predict grain yield and plant height

**Fig. 3:**
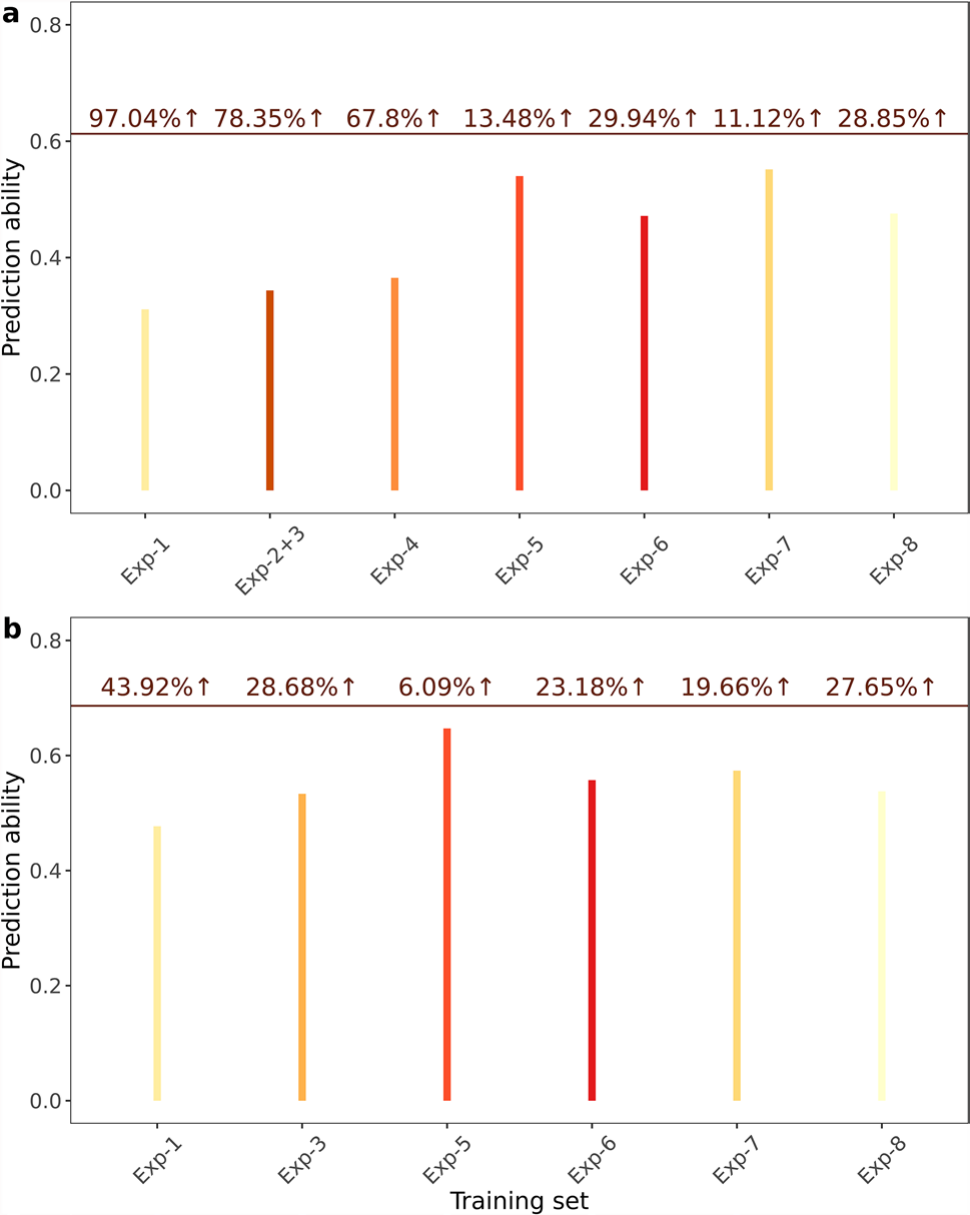
Gain in prediction ability when merging data across experimental series (Exp). **a** Grain yield and **b** plant height plots, the X-axis indicates the training set used to predict the Exp-PRT, and the Y-axis shows the prediction ability. The brown line indicates the prediction abilities with the integrated training set. The numbers above the line refer to the percentage of improvement in the prediction ability due to use of integrated training set compared to the corresponding single experimental series (for grain yield Exp-2 was not used for the comparison due to its small size, instead we used Exp-2 + 3)

The prediction ability based on a single experimental series for the Exp-PRT genotypes ranged between 0.31 (Exp-1) to 0.55 (Exp-7) for grain yield and 0.48 (Exp-1) to 0.65 (Exp-5) for plant height (Supplementary Table 8). The integrated big data across the experimental series reached a prediction ability of 0.61 for grain yield and 0.69 for plant height (Supplementary Table 8). The minimum improvement in predictive ability when moving from a single Exp to an integrated big data approach for grain yield was 11.12% for Exp-7 and the maximum was 97.04% for Exp-1 (Fig. 3a). For plant height, the improvement ranged from 6.09% for Exp-5 to 43.92% for Exp-1 (Fig. 3b).

### Increasing the sample size relative to the diversity of the underlying populations enhances the prediction ability

**Fig. 4:**
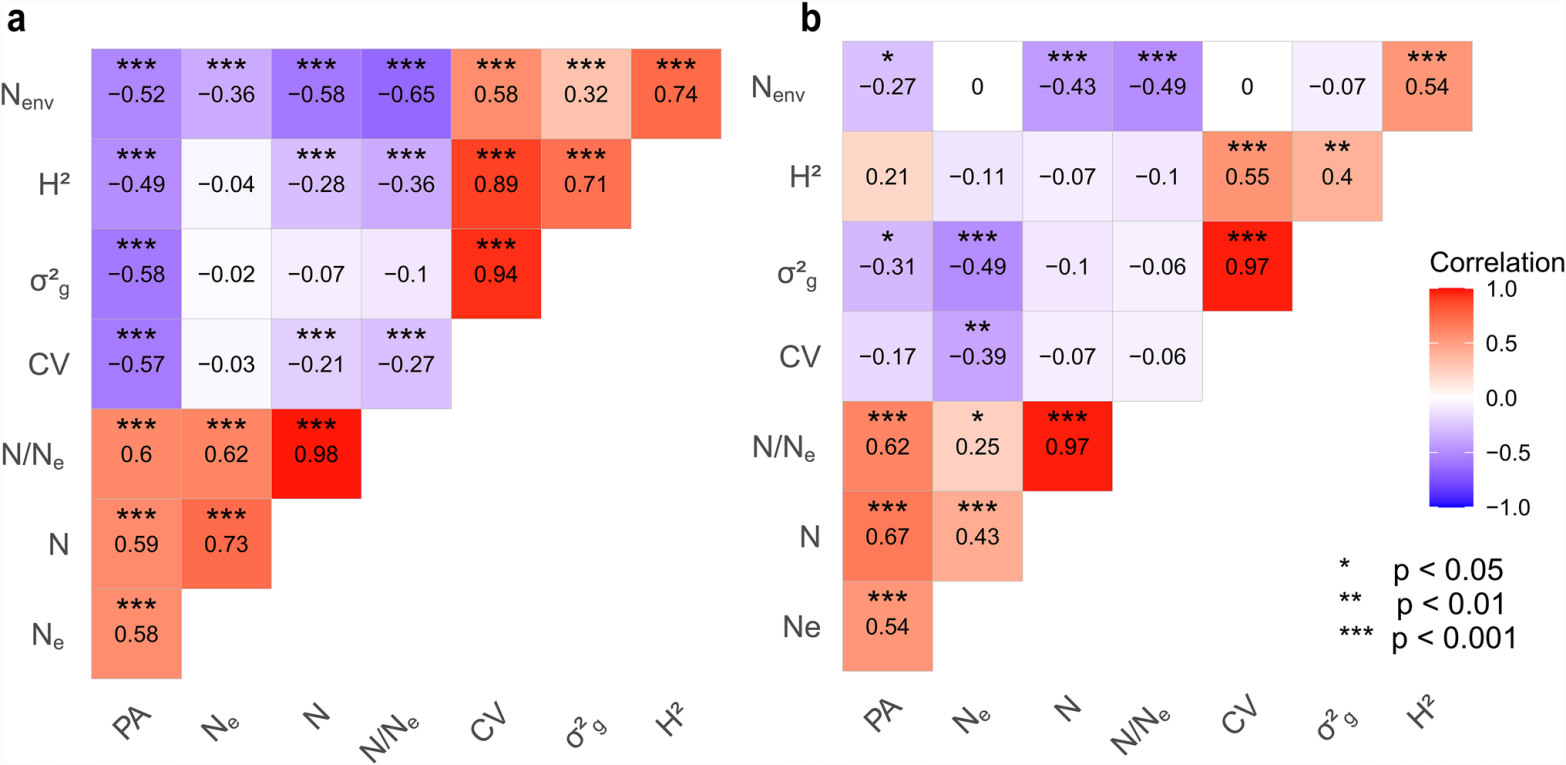
Pearson correlation values between prediction ability (PA) of **a** grain yield and **b** plant height with the effective population size (N_e_), training set size (N), ratio of training set size to the effective population size (N/N_e_), coefficient of variation of BLUPs (CV), genotypic variance (σ^2^_g_), broad-sense heritability (H^2^), and the average number of environments genotypes tested (N_env_), and the. The number of the *marks in colored tiles indicate the significance at respective thresholds

There was a strong positive association between prediction ability and N/N_e_, i.e. the ratio of the size of the training population and the effective population size, with Pearson moment correlation coefficients of r = 0.60 (*p* < 0.001) for grain yield and r = 0.62 (*p* < 0.001) for plant height (Fig. 4a-b). The H^2^ values exhibited positive correlation (r = 0.21) with the prediction ability for plant height but a significant negative correlation (r = − 0.49; *p* < 0.001) was observed for grain yield (Fig. 4a-b). The correlations between the prediction ability for grain yield and CV (r = − 0.57; *p* < 0.001), σ^2^_g_ (r = − 0.58; *p* < 0.001), and N_env_ (r = − 0.52; *p* < 0.001) were significantly negative, while the correlation between the prediction ability for plant height and σ^2^_g_ (r = −0.31; *p* < 0.05) was significantly negative as well (Fig. 4a–b). Nevertheless, the correlation between the prediction ability for plant height and both N_env_ (r = 0) and CV (r = − 0.17) was not statistically significant and is relatively weak (Fig. 4b).

### Quality filtering for missing values and minor allele frequencies improves the prediction ability

**Fig. 5:**
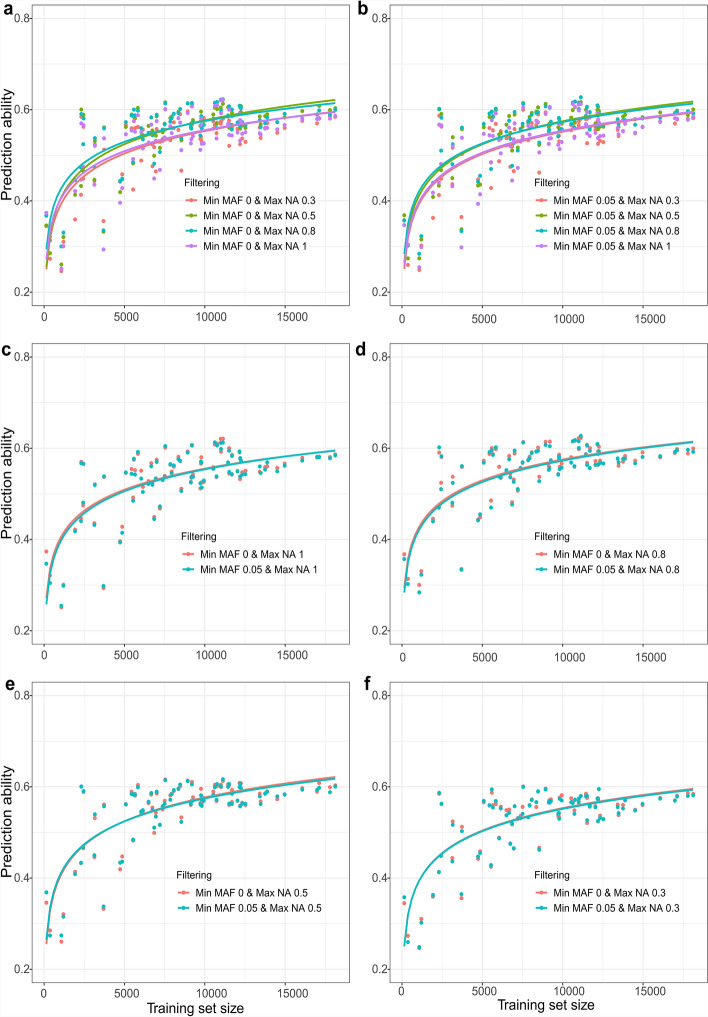
For grain yield, a-b Prediction ability vs Training set size at different NA thresholds and **a** no minor allele filtering (Min MAF 0), **b** minor allele filtering at 5% (Min MAF 0.05). **c**–**f** Prediction ability vs Training set size at different minor allele frequency thresholds and missing values allowed per marker **c** up to 100%, **d** up to 80%, **e** up to 50%, and **f** up to 30%. The colored smoothing lines indicate respective minor allele frequency and missing value filtering thresholds employed and drawn using the formula y ~ a + b * log(x)

For large training populations, filtering out markers with > 50% missing values resulted in higher prediction abilities independent of filtering for minor allele frequency (Supplementary Table 9, Supplementary Table 10). For smaller training sets, removing markers with more than 80% missing values for grain yield and for plant height resulted in higher prediction abilities (Fig. 5a–b, Supplementary Fig. 3a-b). Regardless of the threshold for missing values and the size of the training set, filtering out minor alleles (at 5%) did not lead to better prediction than including minor alleles for either trait (Fig. 5c–f, Supplementary Fig. 3c–f). Thus, our data suggests the advantages of careful quality filtering.

### The inclusion of epistasis improves the prediction ability given the training set size

**Fig. 6:**
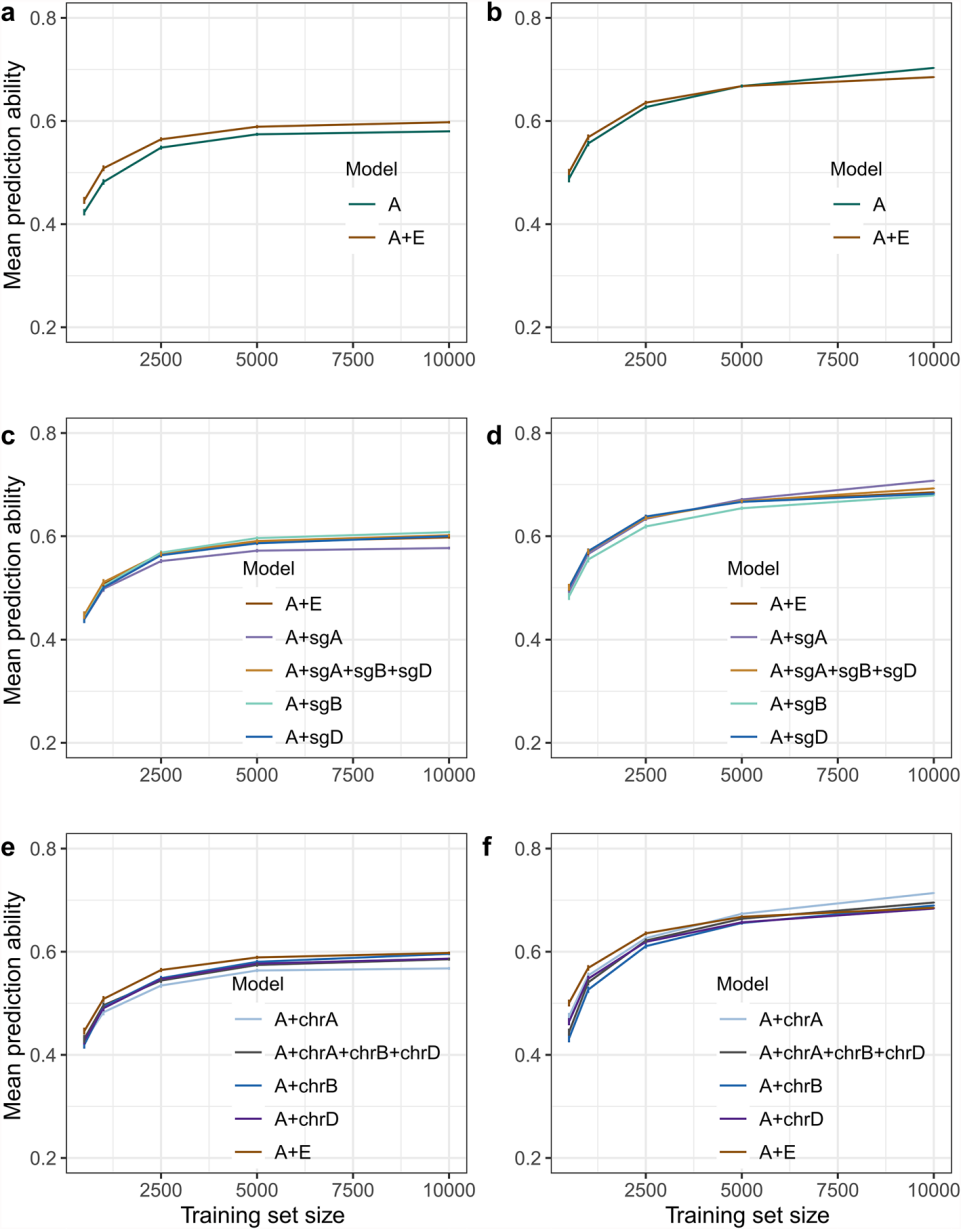
In all the plots, X-axis indicates the training set size and Y-axis indicate the mean prediction ability. **a**–**b** comparison between the additive model (A) and additive plus epistasis model (A + E) for **a** grain yield and **b** plant height. **c**–**d** comparison between the additive plus epistasis model (A + E) and additive plus subgenome epistasis models (A + sgA, sgB, sgD) for **c** grain yield and **d** plant height. **e**–**f** comparison between the additive plus epistasis model (A + E) and additive plus chromosome epistasis models (A + chrA, chrB, chrD) for **e** grain yield and **f** plant height. The color of the lines indicates the different models used, and at each point standard error is indicated for over 100 runs

The incorporation of genome-wide epistasis effects alongside additive effects enhances the prediction ability compared to the sole inclusion of additive effects and the magnitude of this improvement diminishes with the expansion of the training set size for both traits (Fig. 6a-b). It was observed that fitting epistasis within subgenome A for grain yield and subgenome B for plant height consistently demonstrated inferior performance compared to fitting genome-wide epistasis. Fitting the within subgenome B epistasis alone as well as fitting epistasis within subgenome A, B, and D together yielded higher prediction abilities compared to fitting genome-wide epistasis for grain yield. But for plant height, fitting the within subgenome A epistasis alone as well as fitting epistasis within subgenome A, B, and D together yielded higher prediction abilities compared to fitting genome-wide epistasis at larger training set size (Fig. 6c-d). However, none of the chromosome-level epistasis models outperforms the genome-wide epistasis model for grain yield and for plant height epistasis within chromosome A, and fitting epistasis within chromosome A, B, and D together produced higher prediction abilities compared to fitting genome-wide epistasis (Fig. 6e-f).

## Discussion

Zhao et al. (2021) and Lell et al. (2025) demonstrated that combining small to medium datasets from public and private breeding programs improved the prediction ability for wheat hybrids and inbred lines. However, the test sets used were drawn from the combined dataset, limiting the generalizability of their results. In this study, we expanded Lell et al.'s dataset to 18,000 genotypes and validated the potential of big data using the Exp-PRT as a test set. The Exp-PRT contains varieties from different breeding companies and were tested at many locations representing diverse environments in Germany (Supplementary Fig. 1). Our validation yielded reliable and broadly applicable results, with a focus on genomic selection for improved varieties. However, we did not explore the potential of the comprehensive training data for parental selection, as this would require detailed information about the underlying pedigree of the inbred lines included (Rembe et al. 2022).

We found that the integrated training set improved the prediction ability by up to 97% for grain yield and 44% for plant height in the Exp-PRT dataset; outperforming individual training sets (Fig. 3a-b). Furthermore, it was observed that no dataset for both traits exhibited a prediction ability comparable to that of the integrated dataset (Fig. 3a-b). This highlights the power of big data to enhance genomic prediction and accelerate genetic gain compared to phenotypic selection (Heffner et al. 2010; Zhang et al. 2019). However, data sharing between the companies remains a challenge due to confidentiality concerns. A well-defined data-sharing ecosystem is essential to overcome this, along with incorporating multi-omic and environmental data, high-throughput phenotyping, and AI-driven genomic models for further improvement (Xu et al. 2022; Wang et al. 2023; Alemu et al. 2024; Kaushal et al. 2024).

For grain yield, prediction ability plateaus at around 5000 genotypes in our study (Fig. 6a). Similarly, Lell et al. (2025) observed a plateau at ~ 4000 genotypes using 9,000 genotypes. With 18,000 genotypes, the plateau shifts higher, indicating that saturation depends on training set size. This trend is specific to the Central European winter wheat pool; more diverse panels may plateau at different points. Notably, this pattern reflects EGBLUP performance, whereas AI models like CNNs may require even larger datasets (Lell et al. 2025). As increased training size shows no negative impact, we recommend using the full dataset to maximize prediction ability.

We also examined the parameters of the cumulative datasets that drive the prediction ability. The strong associations between the prediction ability and the parameters training set size (N), effective population size (N_e_), and N/N_e_ align with previous studies (Habier et al. 2007; Lorenzana and Bernardo 2009; Meuwissen 2009; Norman et al. 2018; Zhao et al. 2021; and Lell et al. 2025) and justify the superiority of integrated training sets. However, the observed negative correlation between the heritability and its positively associated parameters (CV, N_env_, σ^2^_g_) with the prediction ability for grain yield contradicts previous findings (Combs and Bernardo 2013; Lian et al. 2014; Zhang et al. 2017). This can be explained by the confounding genotype-by-environment interactions observed in the historical experimental series Exp-1 to 4 and the presence of plant genetic resources in Exp-3. Since these experimental series happen to have higher values for the H^2^, CV, N_env_, and σ^2^_g_, their combination with the rest of the datasets inflates these parameters and decreases the prediction ability (Dawson et al. 2013; Jarquín et al. 2014; Berro et al. 2019; Rogers and Holland 2022) compared to the other combinations, leading to negative correlations (Supplementary Table 12). Additionally, datasets with lower H^2^ but larger sizes show higher prediction ability and lead to negative correlations (Daetwyler et al. 2008) (Supplementary Table 12). Furthermore, the negative correlation between the prediction ability and the other parameters (CV, N_env_, and σ^2^_g_) with regard to plant height can be attributed to Exp-1 and Exp-3 with their divergent parameter values. Consequently, when these datasets are merged with the other datasets, the resulting combined datasets exhibit inflated or deflated values of these parameters, resulting in negative correlations (Supplementary Table 12). In summary, increasing the size of the dataset relative to diversity may enhance prediction ability, although correlations should be interpreted with caution.

The handling of missing values and minor alleles in heterogeneous SNP data is crucial for maintaining high genotypic data quality and ensuring optimal prediction ability. Our analysis of four missing value thresholds and two minor allele frequency thresholds (Supplementary Table 9) demonstrated that the inclusion of minor alleles does not harm prediction ability. In fact, the inclusion of minor alleles can enhance prediction ability, particularly when the training set size is substantial (Fig. 5c-f, Supplementary Fig. 3c-f), due to the increased power to detect QTL associated with the minor alleles (Zakieh et al. 2023; Lell et al. 2024). Additionally, our findings indicate that excluding low-quality markers by strict filtering (30%) does not enhance the prediction ability. The potential reason for this could be due to uneven marker genome coverage (Daetwyler et al. 2008), insufficient genotype differentiation, or removal of markers linked to QTLs (Ali et al. 2020). Conversely, using all markers without filtering also decreases the overall quality of the integrated SNP dataset (Ali et al. 2020). Therefore, we recommend a threshold of 50% missing values as a balance between marker number and quality to ensure even genome coverage (Fig. 5a-b and Supplementary Fig. 3a-b). These guidelines apply specifically to quality control of integrated SNP datasets with heterogeneous marker density and genotyping platforms.

As the application of theoretical quantitative genetics assumptions such as random mating and Hardy–Weinberg equilibrium is often violated in breeding populations, the use of classical broad-sense heritability as a benchmark can lead to biased estimations of the empirical upper limit of predictive ability (Bernardo 2020). Therefore, in this study broad-sense heritability was defined in practical terms, and its square root was used as an empirical approximation of the maximum achievable prediction ability. With the availability of big data, we now achieved a predictive power 0.61 for grain yield, which is highly promising. Given the high heritability of Exp-PRT (> 0.96), the genetic variance explained by the predicted value is around 36%. This indicates that about 60% of the genetic variance remains unexplained, highlighting the issue of missing heritability (Manolio et al. 2009). A wide range of explanations has been proposed to explain missing heritability, including factors such as lack of modelling non-additive effects, inadequate population size, uneven genome coverage, rare genetic variants, undetected copy number variation effects and over-estimated heritability (Clarke and Cooper 2010; Park et al. 2010; Visscher et al. 2006; Bodmer and Tomlinson 2010; Forer et al. 2010; Makowsky et al. 2011). In light of this, we considered different epistasis models to explore hidden genetic mechanisms and assess whether they can help to explain any of the 60% of heritability. It is important to note that the epistatic models employed in this study are non-orthogonal, which can potentially introduce bias in the estimation of additive and epistatic variance components (Raffo et al. 2022). However, non-orthogonal models can achieve predictive abilities comparable to those of orthogonal models (Jiang and Reif 2015). Given that our primary aim was to evaluate the contribution of epistasis to prediction ability, rather than to obtain precise variance component estimates, we consider the use of non-orthogonal models to be justified in this context.

Interestingly, the impact of epistasis on prediction ability varies across crops and even within the same crop depending on the population and trait under consideration (Crossa et al. 2010; Jiang and Reif 2015; Raffo et al. 2022). This study shows that the improvement in prediction ability resulting from genome-wide epistasis is contingent upon the genetic architecture of the trait and the size of the training population. Predicting polygenic traits such as grain yield benefits more from modelling epistasis than oligogenic such as plant height. However, this improvement decreases as the training population size increases, probably due to fewer spurious linkage disequilibrium (LD) between unlinked markers (Supplementary Table 13). Smaller populations exhibit inflated LD, detecting more interactions and leading to greater prediction improvements (Supplementary Table 13) (Schrauf et al. 2020).

Previously, Santantonio et al. (2019) and Cuevas et al. (2024) suggested that modelling within subgenome and between subgenome epistatic interaction effects can provide similar prediction abilities as modelling genome-wide epistatic interaction effects. In contrast to the models previously described, our findings show that models incorporating within-subgenome epistasis combined with additive effects outperform the genome-wide epistasis model. Furthermore, incorporating chromosome-level interaction effects did not improve prediction ability for grain yield. Further research is needed to explore the role of subgenome and chromosome level interactions in improving prediction ability. Furthermore, predictions based on both additive and epistatic effects should be used to select the best-performing genotypes, but not for selecting superior parents. This is because epistatic effects are not heritable, and predicted values that include both additive and epistatic components may rank genotypes differently compared to those based solely on additive (heritable) effects (Raffo et al. 2022). In summary, it is strongly recommended that epistasis effects be employed in genomic prediction for wheat, regardless of whether they are apparent or real, particularly for polygenic trait predictions for the purpose of varietal release.

## Supplementary Information

Below is the link to the electronic supplementary material.

Supplementary file1 (PDF 1176 KB)
