# Supplementary material for: Harnessing big data for enhanced genome-wide prediction in winter wheat breeding
Source: Theor Appl Genet. 2025 Aug 22;138(9):224. doi: 10.1007/s00122-025-05007-6 (PMC12373547; doi:10.1007/s00122-025-05007-6)
Supplement: Supplementary file 1 — Supplementary file1 (PDF 1176 KB) [file 122_2025_5007_MOESM1_ESM.pdf]

Supplementary materials

Supplementary tables

Supplementary table 1: Overview of the datasets used in this study.

Dataset	Years	Hybrids		Inbreds		Inbred with phenotypic and genotypic data	
		Plant Height	Grain Yield	Plant Height	Grain Yield	Plant Height	Grain Yield
Exp-PRT	2017-22	8	8	90	90	83	83
Exp-1	2009-10	0	0	380	380	371	371
Exp-2	2012-13	0	1604	0	144	0	142
Exp-3	2016-19	3639	5051	469	1099	467	1082
Exp-4	2012-15	0	0	0	4958	0	3703
Exp-5	2020-23	0	0	3211	3211	3173	3173
Exp-6	2020-23	0	0	2998	3906	1718	1939
Exp-7	2020-23	0	0	2880	2918	2297	2335
Exp-8	2020-23	0	0	6097	8394	3392	5548

Supplementary table 2: Training sets used to study the associated parameters with the prediction ability for the trait grain yield.

Training Set	Trait	Size
Exp-1	Grain Yield	369
Exp-2	Grain Yield	140
Exp-3	Grain Yield	1073
Exp-2 + Exp-3	Grain Yield	1202
Exp-4	Grain Yield	3698
Exp-5	Grain Yield	3158
Exp-6	Grain Yield	1929
Exp-7	Grain Yield	2314
Exp-8	Grain Yield	5540
Exp-1 + Exp-5	Grain Yield	3525
Exp-1 + Exp-6	Grain Yield	2298
Exp-1 + Exp-7	Grain Yield	2683
Exp-1 + Exp-8	Grain Yield	5909
Exp-1 + Exp-3	Grain Yield	1417
Exp-1 + Exp-4	Grain Yield	4053
Exp-1 + Exp-2	Grain Yield	504
Exp-5 + Exp-6	Grain Yield	5087
Exp-5 + Exp-7	Grain Yield	5471
Exp-5 + Exp-8	Grain Yield	8698
Exp-5 + Exp-3	Grain Yield	4227

Exp-5 + Exp-4	Grain Yield	6851
Exp-5 + Exp-2	Grain Yield	3298
Exp-6 + Exp-7	Grain Yield	4243
Exp-6 + Exp-8	Grain Yield	7469
Exp-6 + Exp-3	Grain Yield	3001
Exp-6 + Exp-4	Grain Yield	5626
Exp-6 + Exp-2	Grain Yield	2069
Exp-7 + Exp-8	Grain Yield	7853
Exp-7 + Exp-3	Grain Yield	3386
Exp-7 + Exp-4	Grain Yield	6011
Exp-7 + Exp-2	Grain Yield	2454
Exp-8 + Exp-3	Grain Yield	6611
Exp-8 + Exp-4	Grain Yield	9237
Exp-8 + Exp-2	Grain Yield	5680
Exp-3 + Exp-4	Grain Yield	4732
Exp-3 + Exp-2	Grain Yield	1202
Exp-4 + Exp-2	Grain Yield	3833
Exp-1 + Exp-5 + Exp-6	Grain Yield	5454
Exp-1 + Exp-5 + Exp-7	Grain Yield	5838
Exp-1 + Exp-5 + Exp-8	Grain Yield	9065
Exp-1 + Exp-5 + Exp-3	Grain Yield	4569
Exp-1 + Exp-5 + Exp-4	Grain Yield	7206
Exp-1 + Exp-5 + Exp-2	Grain Yield	3660
Exp-1 + Exp-6 + Exp-7	Grain Yield	4612
Exp-1 + Exp-6 + Exp-8	Grain Yield	7838
Exp-1 + Exp-6 + Exp-3	Grain Yield	3345
Exp-1 + Exp-6 + Exp-4	Grain Yield	5981
Exp-1 + Exp-6 + Exp-2	Grain Yield	2433
Exp-1 + Exp-7 + Exp-8	Grain Yield	8222
Exp-1 + Exp-7 + Exp-3	Grain Yield	3730
Exp-1 + Exp-7 + Exp-4	Grain Yield	6366
Exp-1 + Exp-7 + Exp-2	Grain Yield	2818
Exp-1 + Exp-8 + Exp-3	Grain Yield	6955
Exp-1 + Exp-8 + Exp-4	Grain Yield	9592
Exp-1 + Exp-8 + Exp-2	Grain Yield	6044
Exp-1 + Exp-3 + Exp-4	Grain Yield	5066
Exp-1 + Exp-3 + Exp-2	Grain Yield	1544
Exp-1 + Exp-4 + Exp-2	Grain Yield	4185
Exp-5 + Exp-6 + Exp-7	Grain Yield	7400
Exp-5 + Exp-6 + Exp-8	Grain Yield	10627
Exp-5 + Exp-6 + Exp-3	Grain Yield	6155
Exp-5 + Exp-6 + Exp-4	Grain Yield	8779
Exp-5 + Exp-6 + Exp-2	Grain Yield	5227
Exp-5 + Exp-7 + Exp-8	Grain Yield	11010
Exp-5 + Exp-7 + Exp-3	Grain Yield	6539
Exp-5 + Exp-7 + Exp-4	Grain Yield	9164
Exp-5 + Exp-7 + Exp-2	Grain Yield	5611

Exp-5 + Exp-8 + Exp-3	Grain Yield	9765
Exp-5 + Exp-8 + Exp-4	Grain Yield	12390
Exp-5 + Exp-8 + Exp-2	Grain Yield	8838
Exp-5 + Exp-3 + Exp-4	Grain Yield	7883
Exp-5 + Exp-3 + Exp-2	Grain Yield	4356
Exp-5 + Exp-4 + Exp-2	Grain Yield	6986
Exp-6 + Exp-7 + Exp-8	Grain Yield	9782
Exp-6 + Exp-7 + Exp-3	Grain Yield	5314
Exp-6 + Exp-7 + Exp-4	Grain Yield	7939
Exp-6 + Exp-7 + Exp-2	Grain Yield	4383
Exp-6 + Exp-8 + Exp-3	Grain Yield	8539
Exp-6 + Exp-8 + Exp-4	Grain Yield	11165
Exp-6 + Exp-8 + Exp-2	Grain Yield	7609
Exp-6 + Exp-3 + Exp-4	Grain Yield	6660
Exp-6 + Exp-3 + Exp-2	Grain Yield	3130
Exp-6 + Exp-4 + Exp-2	Grain Yield	5761
Exp-7 + Exp-8 + Exp-3	Grain Yield	8923
Exp-7 + Exp-8 + Exp-4	Grain Yield	11549
Exp-7 + Exp-8 + Exp-2	Grain Yield	7993
Exp-7 + Exp-3 + Exp-4	Grain Yield	7044
Exp-7 + Exp-3 + Exp-2	Grain Yield	3515
Exp-7 + Exp-4 + Exp-2	Grain Yield	6146
Exp-8 + Exp-3 + Exp-4	Grain Yield	10270
Exp-8 + Exp-3 + Exp-2	Grain Yield	6740
Exp-8 + Exp-4 + Exp-2	Grain Yield	9372
Exp-3 + Exp-4 + Exp-2	Grain Yield	4861
Exp-1 + Exp-5 + Exp-6 + Exp-7	Grain Yield	7767
Exp-1 + Exp-5 + Exp-6 + Exp-8	Grain Yield	10994
Exp-1 + Exp-5 + Exp-6 + Exp-3	Grain Yield	6497
Exp-1 + Exp-5 + Exp-6 + Exp-4	Grain Yield	9134
Exp-1 + Exp-5 + Exp-6 + Exp-2	Grain Yield	5589
Exp-1 + Exp-5 + Exp-7 + Exp-8	Grain Yield	11377
Exp-1 + Exp-5 + Exp-7 + Exp-3	Grain Yield	6881
Exp-1 + Exp-5 + Exp-7 + Exp-4	Grain Yield	9519
Exp-1 + Exp-5 + Exp-7 + Exp-2	Grain Yield	5973
Exp-1 + Exp-5 + Exp-8 + Exp-3	Grain Yield	10107
Exp-1 + Exp-5 + Exp-8 + Exp-4	Grain Yield	12745
Exp-1 + Exp-5 + Exp-8 + Exp-2	Grain Yield	9200
Exp-1 + Exp-5 + Exp-3 + Exp-4	Grain Yield	8217
Exp-1 + Exp-5 + Exp-3 + Exp-2	Grain Yield	4696
Exp-1 + Exp-5 + Exp-4 + Exp-2	Grain Yield	7338
Exp-1 + Exp-6 + Exp-7 + Exp-8	Grain Yield	10151
Exp-1 + Exp-6 + Exp-7 + Exp-3	Grain Yield	5658
Exp-1 + Exp-6 + Exp-7 + Exp-4	Grain Yield	8294
Exp-1 + Exp-6 + Exp-7 + Exp-2	Grain Yield	4747
Exp-1 + Exp-6 + Exp-8 + Exp-3	Grain Yield	8883
Exp-1 + Exp-6 + Exp-8 + Exp-4	Grain Yield	11520

Exp-1 + Exp-6 + Exp-8 + Exp-2	Grain Yield	7973
Exp-1 + Exp-6 + Exp-3 + Exp-4	Grain Yield	6994
Exp-1 + Exp-6 + Exp-3 + Exp-2	Grain Yield	3472
Exp-1 + Exp-6 + Exp-4 + Exp-2	Grain Yield	6113
Exp-1 + Exp-7 + Exp-8 + Exp-3	Grain Yield	9267
Exp-1 + Exp-7 + Exp-8 + Exp-4	Grain Yield	11904
Exp-1 + Exp-7 + Exp-8 + Exp-2	Grain Yield	8357
Exp-1 + Exp-7 + Exp-3 + Exp-4	Grain Yield	7378
Exp-1 + Exp-7 + Exp-3 + Exp-2	Grain Yield	3857
Exp-1 + Exp-7 + Exp-4 + Exp-2	Grain Yield	6498
Exp-1 + Exp-8 + Exp-3 + Exp-4	Grain Yield	10604
Exp-1 + Exp-8 + Exp-3 + Exp-2	Grain Yield	7082
Exp-1 + Exp-8 + Exp-4 + Exp-2	Grain Yield	9724
Exp-1 + Exp-3 + Exp-4 + Exp-2	Grain Yield	5193
Exp-5 + Exp-6 + Exp-7 + Exp-8	Grain Yield	12939
Exp-5 + Exp-6 + Exp-7 + Exp-3	Grain Yield	8467
Exp-5 + Exp-6 + Exp-7 + Exp-4	Grain Yield	11092
Exp-5 + Exp-6 + Exp-7 + Exp-2	Grain Yield	7540
Exp-5 + Exp-6 + Exp-8 + Exp-3	Grain Yield	11693
Exp-5 + Exp-6 + Exp-8 + Exp-4	Grain Yield	14318
Exp-5 + Exp-6 + Exp-8 + Exp-2	Grain Yield	10767
Exp-5 + Exp-6 + Exp-3 + Exp-4	Grain Yield	9811
Exp-5 + Exp-6 + Exp-3 + Exp-2	Grain Yield	6284
Exp-5 + Exp-6 + Exp-4 + Exp-2	Grain Yield	8914
Exp-5 + Exp-7 + Exp-8 + Exp-3	Grain Yield	12076
Exp-5 + Exp-7 + Exp-8 + Exp-4	Grain Yield	14702
Exp-5 + Exp-7 + Exp-8 + Exp-2	Grain Yield	11150
Exp-5 + Exp-7 + Exp-3 + Exp-4	Grain Yield	10195
Exp-5 + Exp-7 + Exp-3 + Exp-2	Grain Yield	6668
Exp-5 + Exp-7 + Exp-4 + Exp-2	Grain Yield	9299
Exp-5 + Exp-8 + Exp-3 + Exp-4	Grain Yield	13421
Exp-5 + Exp-8 + Exp-3 + Exp-2	Grain Yield	9894
Exp-5 + Exp-8 + Exp-4 + Exp-2	Grain Yield	12525
Exp-5 + Exp-3 + Exp-4 + Exp-2	Grain Yield	8012
Exp-6 + Exp-7 + Exp-8 + Exp-3	Grain Yield	10851
Exp-6 + Exp-7 + Exp-8 + Exp-4	Grain Yield	13477
Exp-6 + Exp-7 + Exp-8 + Exp-2	Grain Yield	9922
Exp-6 + Exp-7 + Exp-3 + Exp-4	Grain Yield	8972
Exp-6 + Exp-7 + Exp-3 + Exp-2	Grain Yield	5443
Exp-6 + Exp-7 + Exp-4 + Exp-2	Grain Yield	8074
Exp-6 + Exp-8 + Exp-3 + Exp-4	Grain Yield	12198
Exp-6 + Exp-8 + Exp-3 + Exp-2	Grain Yield	8668
Exp-6 + Exp-8 + Exp-4 + Exp-2	Grain Yield	11300
Exp-6 + Exp-3 + Exp-4 + Exp-2	Grain Yield	6789
Exp-7 + Exp-8 + Exp-3 + Exp-4	Grain Yield	12581
Exp-7 + Exp-8 + Exp-3 + Exp-2	Grain Yield	9052
Exp-7 + Exp-8 + Exp-4 + Exp-2	Grain Yield	11684

Exp-7 + Exp-3 + Exp-4 + Exp-2	Grain Yield	7173
Exp-8 + Exp-3 + Exp-4 + Exp-2	Grain Yield	10399
Exp-1 + Exp-5 + Exp-6 + Exp-7 + Exp-8	Grain Yield	13306
Exp-1 + Exp-5 + Exp-6 + Exp-7 + Exp-3	Grain Yield	8809
Exp-1 + Exp-5 + Exp-6 + Exp-7 + Exp-4	Grain Yield	11447
Exp-1 + Exp-5 + Exp-6 + Exp-7 + Exp-2	Grain Yield	7902
Exp-1 + Exp-5 + Exp-6 + Exp-8 + Exp-3	Grain Yield	12035
Exp-1 + Exp-5 + Exp-6 + Exp-8 + Exp-4	Grain Yield	14673
Exp-1 + Exp-5 + Exp-6 + Exp-8 + Exp-2	Grain Yield	11129
Exp-1 + Exp-5 + Exp-6 + Exp-3 + Exp-4	Grain Yield	10145
Exp-1 + Exp-5 + Exp-6 + Exp-3 + Exp-2	Grain Yield	6624
Exp-1 + Exp-5 + Exp-6 + Exp-4 + Exp-2	Grain Yield	9266
Exp-1 + Exp-5 + Exp-7 + Exp-8 + Exp-3	Grain Yield	12418
Exp-1 + Exp-5 + Exp-7 + Exp-8 + Exp-4	Grain Yield	15057
Exp-1 + Exp-5 + Exp-7 + Exp-8 + Exp-2	Grain Yield	11512
Exp-1 + Exp-5 + Exp-7 + Exp-3 + Exp-4	Grain Yield	10529
Exp-1 + Exp-5 + Exp-7 + Exp-3 + Exp-2	Grain Yield	7008
Exp-1 + Exp-5 + Exp-7 + Exp-4 + Exp-2	Grain Yield	9651
Exp-1 + Exp-5 + Exp-8 + Exp-3 + Exp-4	Grain Yield	13755
Exp-1 + Exp-5 + Exp-8 + Exp-3 + Exp-2	Grain Yield	10234
Exp-1 + Exp-5 + Exp-8 + Exp-4 + Exp-2	Grain Yield	12877
Exp-1 + Exp-5 + Exp-3 + Exp-4 + Exp-2	Grain Yield	8344
Exp-1 + Exp-6 + Exp-7 + Exp-8 + Exp-3	Grain Yield	11195
Exp-1 + Exp-6 + Exp-7 + Exp-8 + Exp-4	Grain Yield	13832
Exp-1 + Exp-6 + Exp-7 + Exp-8 + Exp-2	Grain Yield	10286
Exp-1 + Exp-6 + Exp-7 + Exp-3 + Exp-4	Grain Yield	9306
Exp-1 + Exp-6 + Exp-7 + Exp-3 + Exp-2	Grain Yield	5785
Exp-1 + Exp-6 + Exp-7 + Exp-4 + Exp-2	Grain Yield	8426
Exp-1 + Exp-6 + Exp-8 + Exp-3 + Exp-4	Grain Yield	12532
Exp-1 + Exp-6 + Exp-8 + Exp-3 + Exp-2	Grain Yield	9010
Exp-1 + Exp-6 + Exp-8 + Exp-4 + Exp-2	Grain Yield	11652
Exp-1 + Exp-6 + Exp-3 + Exp-4 + Exp-2	Grain Yield	7121
Exp-1 + Exp-7 + Exp-8 + Exp-3 + Exp-4	Grain Yield	12915
Exp-1 + Exp-7 + Exp-8 + Exp-3 + Exp-2	Grain Yield	9394
Exp-1 + Exp-7 + Exp-8 + Exp-4 + Exp-2	Grain Yield	12036
Exp-1 + Exp-7 + Exp-3 + Exp-4 + Exp-2	Grain Yield	7505
Exp-1 + Exp-8 + Exp-3 + Exp-4 + Exp-2	Grain Yield	10731
Exp-5 + Exp-6 + Exp-7 + Exp-8 + Exp-3	Grain Yield	14004
Exp-5 + Exp-6 + Exp-7 + Exp-8 + Exp-4	Grain Yield	16630
Exp-5 + Exp-6 + Exp-7 + Exp-8 + Exp-2	Grain Yield	13079
Exp-5 + Exp-6 + Exp-7 + Exp-3 + Exp-4	Grain Yield	12123
Exp-5 + Exp-6 + Exp-7 + Exp-3 + Exp-2	Grain Yield	8596
Exp-5 + Exp-6 + Exp-7 + Exp-4 + Exp-2	Grain Yield	11227
Exp-5 + Exp-6 + Exp-8 + Exp-3 + Exp-4	Grain Yield	15349
Exp-5 + Exp-6 + Exp-8 + Exp-3 + Exp-2	Grain Yield	11822
Exp-5 + Exp-6 + Exp-8 + Exp-4 + Exp-2	Grain Yield	14453
Exp-5 + Exp-6 + Exp-3 + Exp-4 + Exp-2	Grain Yield	9940

Exp-5 + Exp-7 + Exp-8 + Exp-3 + Exp-4	Grain Yield	15732
Exp-5 + Exp-7 + Exp-8 + Exp-3 + Exp-2	Grain Yield	12205
Exp-5 + Exp-7 + Exp-8 + Exp-4 + Exp-2	Grain Yield	14837
Exp-5 + Exp-7 + Exp-3 + Exp-4 + Exp-2	Grain Yield	10324
Exp-5 + Exp-8 + Exp-3 + Exp-4 + Exp-2	Grain Yield	13550
Exp-6 + Exp-7 + Exp-8 + Exp-3 + Exp-4	Grain Yield	14509
Exp-6 + Exp-7 + Exp-8 + Exp-3 + Exp-2	Grain Yield	10980
Exp-6 + Exp-7 + Exp-8 + Exp-4 + Exp-2	Grain Yield	13612
Exp-6 + Exp-7 + Exp-3 + Exp-4 + Exp-2	Grain Yield	9101
Exp-6 + Exp-8 + Exp-3 + Exp-4 + Exp-2	Grain Yield	12327
Exp-7 + Exp-8 + Exp-3 + Exp-4 + Exp-2	Grain Yield	12710
Exp-1 + Exp-5 + Exp-6 + Exp-7 + Exp-8 + Exp-3	Grain Yield	14346
Exp-1 + Exp-5 + Exp-6 + Exp-7 + Exp-8 + Exp-4	Grain Yield	16985
Exp-1 + Exp-5 + Exp-6 + Exp-7 + Exp-8 + Exp-2	Grain Yield	13441
Exp-1 + Exp-5 + Exp-6 + Exp-7 + Exp-3 + Exp-4	Grain Yield	12457
Exp-1 + Exp-5 + Exp-6 + Exp-7 + Exp-3 + Exp-2	Grain Yield	8936
Exp-1 + Exp-5 + Exp-6 + Exp-7 + Exp-4 + Exp-2	Grain Yield	11579
Exp-1 + Exp-5 + Exp-6 + Exp-8 + Exp-3 + Exp-4	Grain Yield	15683
Exp-1 + Exp-5 + Exp-6 + Exp-8 + Exp-3 + Exp-2	Grain Yield	12162
Exp-1 + Exp-5 + Exp-6 + Exp-8 + Exp-4 + Exp-2	Grain Yield	14805
Exp-1 + Exp-5 + Exp-6 + Exp-3 + Exp-4 + Exp-2	Grain Yield	10272
Exp-1 + Exp-5 + Exp-7 + Exp-8 + Exp-3 + Exp-4	Grain Yield	16066
Exp-1 + Exp-5 + Exp-7 + Exp-8 + Exp-3 + Exp-2	Grain Yield	12545
Exp-1 + Exp-5 + Exp-7 + Exp-8 + Exp-4 + Exp-2	Grain Yield	15189
Exp-1 + Exp-5 + Exp-7 + Exp-3 + Exp-4 + Exp-2	Grain Yield	10656
Exp-1 + Exp-5 + Exp-8 + Exp-3 + Exp-4 + Exp-2	Grain Yield	13882
Exp-1 + Exp-6 + Exp-7 + Exp-8 + Exp-3 + Exp-4	Grain Yield	14843
Exp-1 + Exp-6 + Exp-7 + Exp-8 + Exp-3 + Exp-2	Grain Yield	11322
Exp-1 + Exp-6 + Exp-7 + Exp-8 + Exp-4 + Exp-2	Grain Yield	13964
Exp-1 + Exp-6 + Exp-7 + Exp-3 + Exp-4 + Exp-2	Grain Yield	9433
Exp-1 + Exp-6 + Exp-8 + Exp-3 + Exp-4 + Exp-2	Grain Yield	12659
Exp-1 + Exp-7 + Exp-8 + Exp-3 + Exp-4 + Exp-2	Grain Yield	13042
Exp-5 + Exp-6 + Exp-7 + Exp-8 + Exp-3 + Exp-4	Grain Yield	17660
Exp-5 + Exp-6 + Exp-7 + Exp-8 + Exp-3 + Exp-2	Grain Yield	14133
Exp-5 + Exp-6 + Exp-7 + Exp-8 + Exp-4 + Exp-2	Grain Yield	16765
Exp-5 + Exp-6 + Exp-7 + Exp-3 + Exp-4 + Exp-2	Grain Yield	12252
Exp-5 + Exp-6 + Exp-8 + Exp-3 + Exp-4 + Exp-2	Grain Yield	15478
Exp-5 + Exp-7 + Exp-8 + Exp-3 + Exp-4 + Exp-2	Grain Yield	15861
Exp-6 + Exp-7 + Exp-8 + Exp-3 + Exp-4 + Exp-2	Grain Yield	14638
Exp-1 + Exp-5 + Exp-6 + Exp-7 + Exp-8 + Exp-3 + Exp-4	Grain Yield	17994
Exp-1 + Exp-5 + Exp-6 + Exp-7 + Exp-8 + Exp-3 + Exp-2	Grain Yield	14473
Exp-1 + Exp-5 + Exp-6 + Exp-7 + Exp-8 + Exp-4 + Exp-2	Grain Yield	17117
Exp-1 + Exp-5 + Exp-6 + Exp-7 + Exp-3 + Exp-4 + Exp-2	Grain Yield	12584
Exp-1 + Exp-5 + Exp-6 + Exp-8 + Exp-3 + Exp-4 + Exp-2	Grain Yield	15810
Exp-1 + Exp-5 + Exp-7 + Exp-8 + Exp-3 + Exp-4 + Exp-2	Grain Yield	16193
Exp-1 + Exp-6 + Exp-7 + Exp-8 + Exp-3 + Exp-4 + Exp-2	Grain Yield	14970
Exp-5 + Exp-6 + Exp-7 + Exp-8 + Exp-3 + Exp-4 + Exp-2	Grain Yield	17789

Exp-1 + Exp-5 + Exp-6 + Exp-7 + Exp-8 + Exp-3 + Exp-4 + Exp-2	Grain Yield	18121
Exp-1	Plant Height	369
Exp-3	Plant Height	464
Exp-5	Plant Height	3158
Exp-6	Plant Height	1710
Exp-7	Plant Height	2276
Exp-8	Plant Height	3384
Exp-1 + Exp-5	Plant Height	3525
Exp-1 + Exp-6	Plant Height	2079
Exp-1 + Exp-7	Plant Height	2645
Exp-1 + Exp-8	Plant Height	3753
Exp-1 + Exp-3	Plant Height	832
Exp-5 + Exp-6	Plant Height	4868
Exp-5 + Exp-7	Plant Height	5433
Exp-5 + Exp-8	Plant Height	6542
Exp-5 + Exp-3	Plant Height	3621
Exp-6 + Exp-7	Plant Height	3986
Exp-6 + Exp-8	Plant Height	5094
Exp-6 + Exp-3	Plant Height	2174
Exp-7 + Exp-8	Plant Height	5659
Exp-7 + Exp-3	Plant Height	2740
Exp-8 + Exp-3	Plant Height	3848
Exp-1 + Exp-5 + Exp-6	Plant Height	5235
Exp-1 + Exp-5 + Exp-7	Plant Height	5800
Exp-1 + Exp-5 + Exp-8	Plant Height	6909
Exp-1 + Exp-5 + Exp-3	Plant Height	3987
Exp-1 + Exp-6 + Exp-7	Plant Height	4355
Exp-1 + Exp-6 + Exp-8	Plant Height	5463
Exp-1 + Exp-6 + Exp-3	Plant Height	2542
Exp-1 + Exp-7 + Exp-8	Plant Height	6028
Exp-1 + Exp-7 + Exp-3	Plant Height	3108
Exp-1 + Exp-8 + Exp-3	Plant Height	4216
Exp-5 + Exp-6 + Exp-7	Plant Height	7143
Exp-5 + Exp-6 + Exp-8	Plant Height	8252
Exp-5 + Exp-6 + Exp-3	Plant Height	5331
Exp-5 + Exp-7 + Exp-8	Plant Height	8816
Exp-5 + Exp-7 + Exp-3	Plant Height	5896
Exp-5 + Exp-8 + Exp-3	Plant Height	7005
Exp-6 + Exp-7 + Exp-8	Plant Height	7369
Exp-6 + Exp-7 + Exp-3	Plant Height	4450
Exp-6 + Exp-8 + Exp-3	Plant Height	5558
Exp-7 + Exp-8 + Exp-3	Plant Height	6123
Exp-1 + Exp-5 + Exp-6 + Exp-7	Plant Height	7510
Exp-1 + Exp-5 + Exp-6 + Exp-8	Plant Height	8619
Exp-1 + Exp-5 + Exp-6 + Exp-3	Plant Height	5697
Exp-1 + Exp-5 + Exp-7 + Exp-8	Plant Height	9183
Exp-1 + Exp-5 + Exp-7 + Exp-3	Plant Height	6262

Exp-1 + Exp-5 + Exp-8 + Exp-3	Plant Height	7371
Exp-1 + Exp-6 + Exp-7 + Exp-8	Plant Height	7738
Exp-1 + Exp-6 + Exp-7 + Exp-3	Plant Height	4818
Exp-1 + Exp-6 + Exp-8 + Exp-3	Plant Height	5926
Exp-1 + Exp-7 + Exp-8 + Exp-3	Plant Height	6491
Exp-5 + Exp-6 + Exp-7 + Exp-8	Plant Height	10526
Exp-5 + Exp-6 + Exp-7 + Exp-3	Plant Height	7606
Exp-5 + Exp-6 + Exp-8 + Exp-3	Plant Height	8715
Exp-5 + Exp-7 + Exp-8 + Exp-3	Plant Height	9279
Exp-6 + Exp-7 + Exp-8 + Exp-3	Plant Height	7833
Exp-1 + Exp-5 + Exp-6 + Exp-7 + Exp-8	Plant Height	10893
Exp-1 + Exp-5 + Exp-6 + Exp-7 + Exp-3	Plant Height	7972
Exp-1 + Exp-5 + Exp-6 + Exp-8 + Exp-3	Plant Height	9081
Exp-1 + Exp-5 + Exp-7 + Exp-8 + Exp-3	Plant Height	9645
Exp-1 + Exp-6 + Exp-7 + Exp-8 + Exp-3	Plant Height	8201
Exp-5 + Exp-6 + Exp-7 + Exp-8 + Exp-3	Plant Height	10989
Exp-1 + Exp-5 + Exp-6 + Exp-7 + Exp-8 + Exp-3	Plant Height	11355

Supplementary Table 3: Size and Net effective population size (N_e) of each single and combined dataset (BigData).

**Dataset**	**Size**	**N_e**
BigData	18210	91.2126
Exp-PRT	83	52.3472
Exp-1	371	37.0558
Exp-2	142	54.0334
Exp-3	1082	56.2251
Exp-4	3703	64.5303
Exp-5	3173	68.2500
Exp-6	1941	77.1549
Exp-7	2335	43.3274
Exp-8	5552	61.9180

Supplementary Table 4: The mean and standard deviation (SD) of the Rogers' distance between the genotypes of each experimental series.

**Dataset**	**Mean**	**SD**
Exp-PRT	0.3557	0.0336
Exp-1	0.3563	0.0453
Exp-2	0.3566	0.0354
Exp-3	0.3685	0.0362
Exp-4	0.3455	0.0307
Exp-5	0.3299	0.0295
Exp-6	0.3369	0.0293
Exp-7	0.3259	0.0389
Exp-8	0.3277	0.0322

Supplementary Table 5: The mean and standard deviation (SD) of the Rogers' distance between the genotypes of the experimental series pairs except with the Exp-PRT.

Dataset	Mean	SD
Exp-1 & Exp-2	0.3610	0.0363
Exp-1 & Exp-3	0.3710	0.0362
Exp-1 & Exp-4	0.3589	0.0307
Exp-1 & Exp-5	0.3680	0.0263
Exp-1 & Exp-6	0.3657	0.0247
Exp-1 & Exp-7	0.3619	0.0265
Exp-1 & Exp-8	0.3602	0.0263
Exp-2 & Exp-3	0.3697	0.0354
Exp-2 & Exp-4	0.3579	0.0285
Exp-2 & Exp-5	0.3653	0.0251
Exp-2 & Exp-6	0.3634	0.0234
Exp-2 & Exp-7	0.3580	0.0255
Exp-2 & Exp-8	0.3577	0.0243
Exp-3 & Exp-5	0.3729	0.0302
Exp-3 & Exp-6	0.3699	0.0295
Exp-3 & Exp-7	0.3655	0.0317
Exp-3 & Exp-8	0.3655	0.0310
Exp-4 & Exp-3	0.3674	0.0326
Exp-4 & Exp-5	0.3562	0.0245
Exp-4 & Exp-6	0.3573	0.0214
Exp-4 & Exp-7	0.3513	0.0237
Exp-4 & Exp-8	0.3522	0.0219
Exp-5 & Exp-7	0.3489	0.0256
Exp-5 & Exp-8	0.3522	0.0213
Exp-6 & Exp-5	0.3574	0.0203
Exp-6 & Exp-7	0.3430	0.0253
Exp-6 & Exp-8	0.3424	0.0231
Exp-7 & Exp-8	0.3345	0.0283

Supplementary Table 6: The mean and standard deviation (SD) of the Rogers distance between the genotypes of the Exp-PRT & other experimental series.

Dataset	Mean	SD
Exp-PRT & Exp-1	0.3681	0.0298
Exp-PRT & Exp-2	0.3645	0.0294
Exp-PRT & Exp-4	0.3709	0.0339
Exp-PRT & Exp-3	0.3578	0.0281
Exp-PRT & Exp-6	0.3545	0.0301
Exp-PRT & Exp-5	0.3535	0.0266
Exp-PRT & Exp-7	0.3469	0.0338
Exp-PRT & Exp-8	0.3498	0.0283

Supplementary Table 7: The genotypic variance (σ_g^2), broad-sense heritability (H^2), and the average number of environments (N_{env}) within and across the experimental series for the grain yield and plant height.

Dataset	Trait	σ^2_g	H^2	N_{env}
Exp-PRT	grain yield	10.3248	0.9586	39.2410
Exp-1	grain yield	22.9110	0.8857	8.0216
Exp-2	grain yield	14.7569	0.7786	9.9930
Exp-3	grain yield	51.4174	0.9536	9.9030
Exp-4	grain yield	28.0244	0.8313	4.4086
Exp-5	grain yield	19.1604	0.7620	5.4211
Exp-6	grain yield	11.5233	0.7063	5.0877
Exp-7	grain yield	12.5100	0.7342	5.3820
Exp-8	grain yield	20.5019	0.6071	2.2655
BigData	grain yield	22.0955	0.7957	4.7733
Exp-PRT	plant height	13.9163	0.9807	33.7952
Exp-1	plant height	80.9622	0.9861	8.0216
Exp-3	plant height	8.5396	0.9274	19.5910
Exp-5	plant height	26.7480	0.9294	5.5906
Exp-6	plant height	16.5103	0.7608	2.5175
Exp-7	plant height	21.1575	0.8866	3.7562
Exp-8	plant height	34.3320	0.8194	1.8287
BigData	plant height	27.4630	0.9147	4.5309

Supplementary Table 8: The prediction abilities obtained with single experimental series and integrated experimental series (BigData) as training sets for the traits grain yield and plant height of the Exp-PRT dataset along with their sizes.

Trait	Training Set	Test Set	Training Set Size	Test Set Size	Prediction Ability
grain yield	Exp-1	Exp-PRT	369	83	0.3110
grain yield	Exp-2+3	Exp-PRT	1202	83	0.3436
grain yield	Exp-4	Exp-PRT	3698	83	0.3652
grain yield	Exp-5	Exp-PRT	3158	83	0.5400
grain yield	Exp-6	Exp-PRT	1929	83	0.4716
grain yield	Exp-7	Exp-PRT	2314	83	0.5515
grain yield	Exp-8	Exp-PRT	5540	83	0.4756
grain yield	BigData	Exp-PRT	18121	83	0.6128
plant height	Exp-1	Exp-PRT	369	83	0.4770
plant height	Exp-3	Exp-PRT	464	83	0.5335
plant height	Exp-5	Exp-PRT	3158	83	0.6471
plant height	Exp-6	Exp-PRT	1710	83	0.5573
plant height	Exp-7	Exp-PRT	2276	83	0.5737
plant height	Exp-8	Exp-PRT	3384	83	0.5378
plant height	BigData	Exp-PRT	11355	83	0.6865

Supplementary Table 9: Overview of SNP matrices used to study the MAF and missing value filtering thresholds.

SNP Type	Min MAF	Max NA	Markers
SNP 1	0	<30 %	2555
SNP 2	<5 %	<30 %	2391
SNP 3	0	<50 %	9566
SNP 4	<5 %	<50 %	8505
SNP 5	0	<80 %	15316
SNP 6	<5 %	<80 %	13105
SNP 7	0	100 %	29427
SNP 8	<5 %	100 %	21752

Supplementary Table 10: Training sets used to study the MAF and missing value filtering thresholds.

Training Set	Trait	Size
Exp-2	Grain Yield	140
Exp-1	Grain Yield	369
Exp-3	Grain Yield	1073
Exp-3 + Exp-2	Grain Yield	1202
Exp-6	Grain Yield	1929
Exp-1 + Exp-6	Grain Yield	2298
Exp-7	Grain Yield	2314
Exp-1 + Exp-2 + Exp-6	Grain Yield	2433
Exp-2 + Exp-7	Grain Yield	2454
Exp-3 + Exp-2 + Exp-6	Grain Yield	3130
Exp-5	Grain Yield	3158
Exp-4	Grain Yield	3698
Exp-1 + Exp-3 + Exp-7	Grain Yield	3730
Exp-4 + Exp-3	Grain Yield	4732
Exp-4 + Exp-3 + Exp-2	Grain Yield	4861
Exp-5 + Exp-6	Grain Yield	5087
Exp-3 + Exp-2 + Exp-7 + Exp-6	Grain Yield	5443
Exp-8	Grain Yield	5540
Exp-1 + Exp-5 + Exp-2 + Exp-6	Grain Yield	5589
Exp-1 + Exp-3 + Exp-7 + Exp-6	Grain Yield	5658
Exp-1 + Exp-5 + Exp-7	Grain Yield	5838
Exp-1 + Exp-2 + Exp-8	Grain Yield	6044
Exp-4 + Exp-2 + Exp-7	Grain Yield	6146
Exp-1 + Exp-4 + Exp-2 + Exp-7	Grain Yield	6498
Exp-5 + Exp-3 + Exp-2 + Exp-7	Grain Yield	6668
Exp-3 + Exp-2 + Exp-8	Grain Yield	6740
Exp-4 + Exp-5	Grain Yield	6851
Exp-1 + Exp-3 + Exp-8	Grain Yield	6955
Exp-1 + Exp-5 + Exp-3 + Exp-2 + Exp-7	Grain Yield	7008

Exp-1 + Exp-4 + Exp-5	Grain Yield	7206
Exp-1 + Exp-4 + Exp-3 + Exp-7	Grain Yield	7378
Exp-1 + Exp-4 + Exp-3 + Exp-2 + Exp-7	Grain Yield	7505
Exp-5 + Exp-2 + Exp-7 + Exp-6	Grain Yield	7540
Exp-7 + Exp-8	Grain Yield	7853
Exp-4 + Exp-7 + Exp-6	Grain Yield	7939
Exp-4 + Exp-2 + Exp-7 + Exp-6	Grain Yield	8074
Exp-1 + Exp-2 + Exp-7 + Exp-8	Grain Yield	8357
Exp-5 + Exp-3 + Exp-7 + Exp-6	Grain Yield	8467
Exp-3 + Exp-8 + Exp-6	Grain Yield	8539
Exp-1 + Exp-5 + Exp-3 + Exp-2 + Exp-7 + Exp-6	Grain Yield	8936
Exp-1 + Exp-4 + Exp-5 + Exp-6	Grain Yield	9134
Exp-4 + Exp-5 + Exp-7	Grain Yield	9164
Exp-1 + Exp-5 + Exp-2 + Exp-8	Grain Yield	9200
Exp-1 + Exp-4 + Exp-5 + Exp-2 + Exp-6	Grain Yield	9266
Exp-1 + Exp-4 + Exp-2 + Exp-8	Grain Yield	9724
Exp-5 + Exp-3 + Exp-8	Grain Yield	9765
Exp-7 + Exp-8 + Exp-6	Grain Yield	9782
Exp-4 + Exp-5 + Exp-3 + Exp-6	Grain Yield	9811
Exp-4 + Exp-5 + Exp-3 + Exp-2 + Exp-6	Grain Yield	9940
Exp-1 + Exp-4 + Exp-5 + Exp-3 + Exp-7	Grain Yield	10529
Exp-5 + Exp-8 + Exp-6	Grain Yield	10627
Exp-1 + Exp-4 + Exp-3 + Exp-2 + Exp-8	Grain Yield	10731
Exp-5 + Exp-2 + Exp-8 + Exp-6	Grain Yield	10767
Exp-1 + Exp-5 + Exp-8 + Exp-6	Grain Yield	10994
Exp-5 + Exp-7 + Exp-8	Grain Yield	11010
Exp-1 + Exp-5 + Exp-2 + Exp-8 + Exp-6	Grain Yield	11129
Exp-1 + Exp-4 + Exp-5 + Exp-7 + Exp-6	Grain Yield	11447
Exp-1 + Exp-5 + Exp-2 + Exp-7 + Exp-8	Grain Yield	11512
Exp-1 + Exp-4 + Exp-8 + Exp-6	Grain Yield	11520
Exp-4 + Exp-2 + Exp-7 + Exp-8	Grain Yield	11684
Exp-5 + Exp-3 + Exp-8 + Exp-6	Grain Yield	11693
Exp-1 + Exp-4 + Exp-2 + Exp-7 + Exp-8	Grain Yield	12036
Exp-1 + Exp-5 + Exp-3 + Exp-2 + Exp-8 + Exp-6	Grain Yield	12162
Exp-4 + Exp-3 + Exp-8 + Exp-6	Grain Yield	12198
Exp-5 + Exp-3 + Exp-2 + Exp-7 + Exp-8	Grain Yield	12205
Exp-4 + Exp-5 + Exp-3 + Exp-2 + Exp-7 + Exp-6	Grain Yield	12252
Exp-4 + Exp-3 + Exp-2 + Exp-8 + Exp-6	Grain Yield	12327
Exp-1 + Exp-4 + Exp-3 + Exp-8 + Exp-6	Grain Yield	12532
Exp-4 + Exp-5 + Exp-3 + Exp-8	Grain Yield	13421
Exp-4 + Exp-7 + Exp-8 + Exp-6	Grain Yield	13477
Exp-1 + Exp-4 + Exp-5 + Exp-3 + Exp-8	Grain Yield	13755
Exp-1 + Exp-4 + Exp-5 + Exp-3 + Exp-2 + Exp-8	Grain Yield	13882
Exp-4 + Exp-3 + Exp-7 + Exp-8 + Exp-6	Grain Yield	14509
Exp-1 + Exp-4 + Exp-3 + Exp-2 + Exp-7 + Exp-8 + Exp-6	Grain Yield	14970
Exp-1 + Exp-4 + Exp-5 + Exp-3 + Exp-7 + Exp-8	Grain Yield	16066
Exp-1 + Exp-4 + Exp-5 + Exp-7 + Exp-8 + Exp-6	Grain Yield	16985

Exp-1 + Exp-4 + Exp-5 + Exp-2 + Exp-7 + Exp-8 + Exp-6	Grain Yield	17117
Exp-4 + Exp-5 + Exp-3 + Exp-2 + Exp-7 + Exp-8 + Exp-6	Grain Yield	17789
Exp-1 + Exp-4 + Exp-5 + Exp-3 + Exp-2 + Exp-7 + Exp-8 + Exp-6	Grain Yield	18121
Exp-1	Plant Height	369
Exp-3	Plant Height	464
Exp-1 + Exp-3	Plant Height	832
Exp-6	Plant Height	1710
Exp-3 + Exp-6	Plant Height	2174
Exp-7	Plant Height	2276
Exp-1 + Exp-3 + Exp-6	Plant Height	2542
Exp-1 + Exp-7	Plant Height	2645
Exp-1 + Exp-3 + Exp-7	Plant Height	3108
Exp-5	Plant Height	3158
Exp-8	Plant Height	3384
Exp-1 + Exp-5	Plant Height	3525
Exp-5 + Exp-3	Plant Height	3621
Exp-1 + Exp-3 + Exp-8	Plant Height	4216
Exp-1 + Exp-7 + Exp-6	Plant Height	4355
Exp-3 + Exp-7 + Exp-6	Plant Height	4450
Exp-5 + Exp-6	Plant Height	4868
Exp-8 + Exp-6	Plant Height	5094
Exp-5 + Exp-3 + Exp-6	Plant Height	5331
Exp-5 + Exp-7	Plant Height	5433
Exp-1 + Exp-8 + Exp-6	Plant Height	5463
Exp-3 + Exp-8 + Exp-6	Plant Height	5558
Exp-7 + Exp-8	Plant Height	5659
Exp-1 + Exp-5 + Exp-7	Plant Height	5800
Exp-5 + Exp-3 + Exp-7	Plant Height	5896
Exp-1 + Exp-3 + Exp-8 + Exp-6	Plant Height	5926
Exp-1 + Exp-7 + Exp-8	Plant Height	6028
Exp-3 + Exp-7 + Exp-8	Plant Height	6123
Exp-1 + Exp-5 + Exp-3 + Exp-7	Plant Height	6262
Exp-1 + Exp-3 + Exp-7 + Exp-8	Plant Height	6491
Exp-5 + Exp-8	Plant Height	6542
Exp-5 + Exp-7 + Exp-6	Plant Height	7143
Exp-7 + Exp-8 + Exp-6	Plant Height	7369
Exp-1 + Exp-5 + Exp-3 + Exp-8	Plant Height	7371
Exp-1 + Exp-5 + Exp-7 + Exp-6	Plant Height	7510
Exp-1 + Exp-5 + Exp-3 + Exp-7 + Exp-6	Plant Height	7972
Exp-1 + Exp-3 + Exp-7 + Exp-8 + Exp-6	Plant Height	8201
Exp-5 + Exp-8 + Exp-6	Plant Height	8252
Exp-1 + Exp-5 + Exp-8 + Exp-6	Plant Height	8619
Exp-5 + Exp-3 + Exp-8 + Exp-6	Plant Height	8715
Exp-1 + Exp-5 + Exp-3 + Exp-8 + Exp-6	Plant Height	9081
Exp-1 + Exp-5 + Exp-7 + Exp-8	Plant Height	9183
Exp-5 + Exp-3 + Exp-7 + Exp-8	Plant Height	9279
Exp-1 + Exp-5 + Exp-7 + Exp-8 + Exp-6	Plant Height	10893

Exp-5 + Exp-3 + Exp-7 + Exp-8 + Exp-6	Plant Height	10989
Exp-1 + Exp-5 + Exp-3 + Exp-7 + Exp-8 + Exp-6	Plant Height	11355

Supplementary Table 11: Training sets used for predicting the grain yield and plant height of Exp-PRT and their trial years, size, and prediction abilities.

Training Set	Years	Test Set	Years	Training Set Size	Test Set Size	Prediction Ability	Trait
Exp-1	2009-10	Exp-PRT	2017-22	369	83	0.3110	Grain Yield
Exp-2	2012-13	Exp-PRT	2017-22	140	83	0.3447	Grain Yield
Exp-3	2016-19	Exp-PRT	2017-22	1073	83	0.2910	Grain Yield
Exp-2+3	2012-19	Exp-PRT	2017-22	1202	83	0.3436	Grain Yield
Exp-4	2012-15	Exp-PRT	2017-22	3698	83	0.3652	Grain Yield
Exp-5	2020-23	Exp-PRT	2017-22	3158	83	0.5400	Grain Yield
Exp-6	2020-23	Exp-PRT	2017-22	1929	83	0.4716	Grain Yield
Exp-7	2020-23	Exp-PRT	2017-22	2314	83	0.5515	Grain Yield
Exp-8	2020-23	Exp-PRT	2017-22	5540	83	0.4756	Grain Yield
Exp-1	2009-10	Exp-PRT	2017-22	369	83	0.4770	Plant Height
Exp-3	2016-19	Exp-PRT	2017-22	464	83	0.5335	Plant Height
Exp-5	2020-23	Exp-PRT	2017-22	3158	83	0.6471	Plant Height
Exp-6	2020-23	Exp-PRT	2017-22	1710	83	0.5573	Plant Height
Exp-7	2020-23	Exp-PRT	2017-22	2276	83	0.5737	Plant Height
Exp-8	2020-23	Exp-PRT	2017-22	3384	83	0.5378	Plant Height

Supplementary Table 12: Datasets and all possible combinations along with their size (N), prediction ability (PA), coefficient of variation (CV), genotypic variance (σ^2_g), heritability (H^2), the average number of environments a genotype tested in (Nenv), effective population size (Ne), and N/Ne ratio for the traits grain yield and plant height.

Dataset	Trait	PA	N	CV	σ^2_g	H^2	N_{env}	N_e	N/N_e
Exp-1	Grain Yield	0.3111	369	4.6558	22.911	0.8857	8.0216	36.845	10.0149
Exp-5	Grain Yield	0.5443	3158	3.8955	19.1604	0.762	5.4211	68.8316	45.8801
Exp-6	Grain Yield	0.4661	1929	3.0174	11.5233	0.7063	5.0877	77.6623	24.8383
Exp-7	Grain Yield	0.5312	2314	3.0287	12.51	0.7342	5.382	43.7653	52.8729
Exp-8	Grain Yield	0.4911	5540	3.2642	20.5019	0.6071	2.2655	61.9484	89.4293
Exp-3	Grain Yield	0.2479	1073	8.3773	51.4174	0.9536	9.903	56.0122	19.1565
Exp-4	Grain Yield	0.3624	3698	4.7479	28.0244	0.8313	4.4086	64.621	57.2260
Exp-2	Grain Yield	0.3542	140	3.5062	14.7569	0.7786	9.993	53.7277	2.6057
Exp-1 + Exp-5	Grain Yield	0.5685	3525	4.0157	19.7674	0.7837	5.6981	68.6001	51.3848
Exp-1 + Exp-6	Grain Yield	0.5239	2298	3.4472	13.9336	0.7625	5.5613	79.4197	28.9349
Exp-1 + Exp-7	Grain Yield	0.5242	2683	3.3531	14.3908	0.7738	5.746	48.7752	55.0075
Exp-1 + Exp-8	Grain Yield	0.5063	5909	3.5597	21.993	0.6716	2.6267	64.7182	91.3035

Exp-1 + Exp-3	Grain Yield	0.3234	1417	7.5779	44.2179	0.9452	9.5943	54.9733	25.7761
Exp-1 + Exp-4	Grain Yield	0.3877	4053	4.7557	27.5782	0.84	4.7551	67.2757	60.2446
Exp-1 + Exp-2	Grain Yield	0.3595	504	4.3194	20.405	0.8563	8.6686	44.2529	11.3891
Exp-5 + Exp-6	Grain Yield	0.5971	5087	3.6866	16.4203	0.7469	5.3029	74.8994	67.9178
Exp-5 + Exp-7	Grain Yield	0.5985	5471	3.5584	16.4308	0.7541	5.4153	60.4646	90.4827
Exp-5 + Exp-8	Grain Yield	0.5891	8698	3.4185	19.2967	0.6754	3.4159	68.3893	127.1836
Exp-5 + Exp-3	Grain Yield	0.5111	4227	5.255	26.212	0.8534	6.5731	68.4183	61.7817
Exp-5 + Exp-4	Grain Yield	0.5189	6851	4.3082	23.2949	0.7967	4.8808	73.4442	93.2817
Exp-5 + Exp-2	Grain Yield	0.5396	3298	3.855	18.8186	0.7608	5.6186	69.6009	47.3844
Exp-6 + Exp-7	Grain Yield	0.6198	4243	3.0797	12.0933	0.7229	5.2583	65.8015	64.4818
Exp-6 + Exp-8	Grain Yield	0.5816	7469	3.281	17.2251	0.6523	2.9984	75.0956	99.4599
Exp-6 + Exp-3	Grain Yield	0.5069	3001	5.1716	24.8696	0.8724	6.8213	77.0271	38.9603
Exp-6 + Exp-4	Grain Yield	0.5005	5626	4.3891	22.0609	0.8048	4.6444	81.2052	69.2813
Exp-6 + Exp-2	Grain Yield	0.5138	2069	2.9688	11.481	0.6992	5.425	80.599	25.6703
Exp-7 + Exp-8	Grain Yield	0.5544	7853	3.195	17.3577	0.6717	3.1919	64.5396	121.6772

Exp-7 + Exp-3	Grain Yield	0.4956	3386	5.0697	24.0179	0.869	6.8256	53.8584	62.8686
Exp-7 + Exp-4	Grain Yield	0.5278	6011	4.1622	21.7647	0.808	4.789	67.7444	88.7306
Exp-7 + Exp-2	Grain Yield	0.5499	2454	2.9967	12.3668	0.7264	5.6509	46.5894	52.6729
Exp-8 + Exp-3	Grain Yield	0.5369	6611	5.0204	29.9456	0.7974	3.514	66.5602	99.3236
Exp-8 + Exp-4	Grain Yield	0.5107	9237	4.145	25.6033	0.7501	3.1244	74.5684	123.8728
Exp-8 + Exp-2	Grain Yield	0.5382	5680	3.0514	18.6752	0.5884	2.4588	63.468	89.4939
Exp-3 + Exp-4	Grain Yield	0.4645	4732	6.0506	34.4112	0.8857	5.7034	71.9627	65.7563
Exp-3 + Exp-2	Grain Yield	0.3203	1202	7.7461	46.5655	0.9454	10.0198	57.88	20.7671
Exp-4 + Exp-2	Grain Yield	0.3925	3833	4.6553	26.9919	0.8239	4.6232	65.9873	58.0869
Exp-1 + Exp-5 + Exp-6	Grain Yield	0.6293	5454	3.7914	17.0373	0.764	5.4901	78.874	69.1483
Exp-1 + Exp-5 + Exp-7	Grain Yield	0.588	5838	3.6606	17.0055	0.7691	5.5829	64.0587	91.1352
Exp-1 + Exp-5 + Exp-8	Grain Yield	0.5997	9065	3.5374	19.8615	0.6992	3.6052	71.1792	127.3546
Exp-1 + Exp-5 + Exp-3	Grain Yield	0.519	4569	5.2379	26.2085	0.8587	6.7303	69.2466	65.9816
Exp-1 + Exp-5 + Exp-4	Grain Yield	0.5208	7206	4.3427	23.3718	0.8049	5.0522	74.8919	96.2187
Exp-1 + Exp-5 + Exp-2	Grain Yield	0.5709	3660	3.9755	19.4366	0.7817	5.8733	69.2858	52.8247

Exp-1 + Exp-6 + Exp-7	Grain Yield	0.5989	4612	3.2866	13.2763	0.7501	5.4806	69.4877	66.3715
Exp-1 + Exp-6 + Exp-8	Grain Yield	0.602	7838	3.4526	18.1675	0.6863	3.2361	77.2252	101.4954
Exp-1 + Exp-6 + Exp-3	Grain Yield	0.5245	3345	5.1755	24.9657	0.8764	7.0065	77.8962	42.9418
Exp-1 + Exp-6 + Exp-4	Grain Yield	0.526	5981	4.4121	22.1795	0.813	4.8651	83.062	72.0065
Exp-1 + Exp-6 + Exp-2	Grain Yield	0.5636	2433	3.382	13.7486	0.7548	5.8323	80.4548	30.2406
Exp-1 + Exp-7 + Exp-8	Grain Yield	0.5558	8222	3.3474	18.164	0.6998	3.4096	66.7775	123.1253
Exp-1 + Exp-7 + Exp-3	Grain Yield	0.518	3730	5.0684	24.1957	0.873	6.9909	56.3838	66.1538
Exp-1 + Exp-7 + Exp-4	Grain Yield	0.5341	6366	4.201	21.8969	0.8153	4.9879	69.8831	91.0950
Exp-1 + Exp-7 + Exp-2	Grain Yield	0.5222	2818	3.3124	14.1682	0.7665	5.9725	50.6028	55.6886
Exp-1 + Exp-8 + Exp-3	Grain Yield	0.5422	6955	5.0183	29.7583	0.8097	3.767	67.982	102.3065
Exp-1 + Exp-8 + Exp-4	Grain Yield	0.5191	9592	4.1845	25.5779	0.7627	3.3184	76.0931	126.0561
Exp-1 + Exp-8 + Exp-2	Grain Yield	0.5358	6044	3.389	20.5182	0.6573	2.8021	65.6754	92.0284
Exp-1 + Exp-3 + Exp-4	Grain Yield	0.4493	5066	5.9586	33.74	0.8878	5.9133	72.7744	69.6124
Exp-1 + Exp-3 + Exp-2	Grain Yield	0.3582	1544	7.1446	41.1013	0.9381	9.7233	56.6108	27.2739
Exp-1 + Exp-4 + Exp-2	Grain Yield	0.4058	4185	4.6729	26.7003	0.8337	4.9439	68.287	61.2855

Exp-5 + Exp-6 + Exp-7	Grain Yield	0.6256	7400	3.5005	15.2205	0.7446	5.3356	73.031	101.3268
Exp-5 + Exp-6 + Exp-8	Grain Yield	0.637	10627	3.4394	17.6447	0.6878	3.723	78.7298	134.9807
Exp-5 + Exp-6 + Exp-3	Grain Yield	0.5921	6155	4.6182	21.7564	0.8246	6.1164	82.1827	74.8941
Exp-5 + Exp-6 + Exp-4	Grain Yield	0.5721	8779	4.1794	20.7288	0.7852	4.9314	85.9747	102.1114
Exp-5 + Exp-6 + Exp-2	Grain Yield	0.598	5227	3.6492	16.2636	0.7452	5.4309	76.8941	67.9766
Exp-5 + Exp-7 + Exp-8	Grain Yield	0.5992	11010	3.3541	17.6573	0.6972	3.8359	70.1331	156.9872
Exp-5 + Exp-7 + Exp-3	Grain Yield	0.567	6539	4.5446	21.4498	0.8251	6.1637	68.0741	96.0571
Exp-5 + Exp-7 + Exp-4	Grain Yield	0.5702	9164	4.0288	20.5651	0.7881	5.015	75.2824	121.7283
Exp-5 + Exp-7 + Exp-2	Grain Yield	0.586	5611	3.5366	16.2767	0.7522	5.5326	62.1467	90.2864
Exp-5 + Exp-8 + Exp-3	Grain Yield	0.5702	9765	4.4028	24.2499	0.7723	4.1366	74.7468	130.6410
Exp-5 + Exp-8 + Exp-4	Grain Yield	0.5699	12390	3.9399	22.6971	0.743	3.7143	81.1327	152.7128
Exp-5 + Exp-8 + Exp-2	Grain Yield	0.5872	8838	3.3816	18.9331	0.6738	3.5218	69.6006	126.9817
Exp-5 + Exp-3 + Exp-4	Grain Yield	0.5316	7883	5.1488	27.3817	0.8445	5.598	78.2622	100.7255
Exp-5 + Exp-3 + Exp-2	Grain Yield	0.5169	4356	5.1544	25.7407	0.8507	6.7036	69.5195	62.6587
Exp-5 + Exp-4 + Exp-2	Grain Yield	0.5038	6986	4.2721	22.9553	0.7945	4.9893	74.2095	94.1389

Exp-6 + Exp-7 + Exp-8	Grain Yield	0.6042	9782	3.2012	15.7183	0.6802	3.5693	74.6629	131.0155
Exp-6 + Exp-7 + Exp-3	Grain Yield	0.5929	5314	4.3613	19.4478	0.8302	6.2061	72.6031	73.1925
Exp-6 + Exp-7 + Exp-4	Grain Yield	0.577	7939	4.0148	19.1657	0.7907	4.8671	80.3032	98.8628
Exp-6 + Exp-7 + Exp-2	Grain Yield	0.6153	4383	3.0459	12.0171	0.7182	5.4133	67.727	64.7157
Exp-6 + Exp-8 + Exp-3	Grain Yield	0.592	8539	4.425	24.0309	0.7799	3.8735	78.9544	108.1510
Exp-6 + Exp-8 + Exp-4	Grain Yield	0.5721	11165	4.0464	22.3395	0.7475	3.4665	84.4784	132.1640
Exp-6 + Exp-8 + Exp-2	Grain Yield	0.6053	7609	3.1579	16.45	0.6416	3.1291	76.1124	99.9706
Exp-6 + Exp-3 + Exp-4	Grain Yield	0.539	6660	5.2306	27.3012	0.8571	5.528	86.7469	76.7751
Exp-6 + Exp-3 + Exp-2	Grain Yield	0.5085	3130	5.0432	24.1825	0.8664	6.9927	78.6107	39.8165
Exp-6 + Exp-4 + Exp-2	Grain Yield	0.5228	5761	4.3035	21.5758	0.7994	4.7816	82.1647	70.1153
Exp-7 + Exp-8 + Exp-3	Grain Yield	0.5639	8923	4.3611	23.6227	0.7838	4.0061	69.2237	128.9009
Exp-7 + Exp-8 + Exp-4	Grain Yield	0.544	11549	3.8951	22.1323	0.753	3.5829	75.9678	152.0249
Exp-7 + Exp-8 + Exp-2	Grain Yield	0.5769	7993	3.104	16.6605	0.662	3.3132	65.5384	121.9590
Exp-7 + Exp-3 + Exp-4	Grain Yield	0.5544	7044	5.1193	26.7486	0.8566	5.6029	74.0131	95.1723
Exp-7 + Exp-3 + Exp-2	Grain Yield	0.5205	3515	4.939	23.4076	0.8634	6.9777	55.4991	63.3344

Exp-7 + Exp-4 + Exp-2	Grain Yield	0.5376	6146	4.1078	21.314	0.8029	4.9142	68.7668	89.3745
Exp-8 + Exp-3 + Exp-4	Grain Yield	0.5397	10270	5.0251	29.7822	0.8136	3.8511	79.0763	129.8746
Exp-8 + Exp-3 + Exp-2	Grain Yield	0.5551	6740	4.8454	28.8326	0.7906	3.657	67.4851	99.8739
Exp-8 + Exp-4 + Exp-2	Grain Yield	0.5317	9372	4.0659	24.8545	0.7439	3.2306	75.2835	124.4894
Exp-3 + Exp-4 + Exp-2	Grain Yield	0.4689	4861	5.9041	33.5192	0.8817	5.8437	72.8118	66.7612
Exp-1 + Exp-5 + Exp-6 + Exp-7	Grain Yield	0.6233	7767	3.5863	15.7287	0.7573	5.4655	75.9981	102.1999
Exp-1 + Exp-5 + Exp-6 + Exp-8	Grain Yield	0.6558	10994	3.5296	18.1229	0.7053	3.8688	81.0932	135.5724
Exp-1 + Exp-5 + Exp-6 + Exp-3	Grain Yield	0.6061	6497	4.6512	22.0101	0.8311	6.2511	83.4953	77.8128
Exp-1 + Exp-5 + Exp-6 + Exp-4	Grain Yield	0.5815	9134	4.2105	20.8994	0.7925	5.0647	87.1438	104.8153
Exp-1 + Exp-5 + Exp-6 + Exp-2	Grain Yield	0.6299	5589	3.7549	16.8742	0.7621	5.61	80.1231	69.7552
Exp-1 + Exp-5 + Exp-7 + Exp-8	Grain Yield	0.5998	11377	3.4411	18.1015	0.7129	3.9731	72.3155	157.3245
Exp-1 + Exp-5 + Exp-7 + Exp-3	Grain Yield	0.5685	6881	4.5741	21.7077	0.8312	6.2884	69.7128	98.7050
Exp-1 + Exp-5 + Exp-7 + Exp-4	Grain Yield	0.5679	9519	4.066	20.7354	0.7948	5.1397	76.6532	124.1827
Exp-1 + Exp-5 + Exp-7 + Exp-2	Grain Yield	0.5935	5973	3.6383	16.8457	0.7671	5.6938	65.2498	91.5405
Exp-1 + Exp-5 + Exp-8 + Exp-3	Grain Yield	0.5803	10107	4.4426	24.4484	0.7826	4.2903	76.2872	132.4862

Exp-1 + Exp-5 + Exp-8 + Exp-4	Grain Yield	0.5695	12745	3.9828	22.8326	0.7527	3.8439	82.3699	154.7289
Exp-1 + Exp-5 + Exp-8 + Exp-2	Grain Yield	0.601	9200	3.5008	19.5095	0.6973	3.7059	72.1355	127.5378
Exp-1 + Exp-5 + Exp-3 + Exp-4	Grain Yield	0.5144	8217	5.1405	27.3398	0.8486	5.7316	78.7999	104.2768
Exp-1 + Exp-5 + Exp-3 + Exp-2	Grain Yield	0.5201	4696	5.147	25.7619	0.8561	6.8499	70.054	67.0340
Exp-1 + Exp-5 + Exp-4 + Exp-2	Grain Yield	0.5249	7338	4.3086	23.0537	0.8028	5.1544	75.5004	97.1915
Exp-1 + Exp-6 + Exp-7 + Exp-8	Grain Yield	0.5975	10151	3.3202	16.3872	0.7011	3.732	76.5254	132.6488
Exp-1 + Exp-6 + Exp-7 + Exp-3	Grain Yield	0.5985	5658	4.4198	19.8914	0.8371	6.353	74.1376	76.3176
Exp-1 + Exp-6 + Exp-7 + Exp-4	Grain Yield	0.571	8294	4.0534	19.4083	0.7978	5.0166	81.8481	101.3341
Exp-1 + Exp-6 + Exp-7 + Exp-2	Grain Yield	0.6091	4747	3.2504	13.168	0.7456	5.6229	70.7424	67.1026
Exp-1 + Exp-6 + Exp-8 + Exp-3	Grain Yield	0.6068	8883	4.4687	24.2334	0.7907	4.0576	80.0253	111.0024
Exp-1 + Exp-6 + Exp-8 + Exp-4	Grain Yield	0.578	11520	4.0789	22.4563	0.7574	3.6175	85.6628	134.4808
Exp-1 + Exp-6 + Exp-8 + Exp-2	Grain Yield	0.6153	7973	3.3475	17.4972	0.6777	3.3587	77.999	102.2193
Exp-1 + Exp-6 + Exp-3 + Exp-4	Grain Yield	0.5376	6994	5.2171	27.2141	0.8605	5.6883	87.1972	80.2090
Exp-1 + Exp-6 + Exp-3 + Exp-2	Grain Yield	0.5249	3472	5.0603	24.3381	0.8711	7.1582	78.9649	43.9689
Exp-1 + Exp-6 + Exp-4 + Exp-2	Grain Yield	0.5365	6113	4.3369	21.7431	0.8084	4.9918	83.7814	72.9637

Exp-1 + Exp-7 + Exp-8 + Exp-3	Grain Yield	0.57	9267	4.3999	23.8323	0.7937	4.1776	70.4234	131.5898
Exp-1 + Exp-7 + Exp-8 + Exp-4	Grain Yield	0.543	11904	3.9342	22.2493	0.762	3.7254	77.2014	154.1941
Exp-1 + Exp-7 + Exp-8 + Exp-2	Grain Yield	0.5703	8357	3.2691	17.5538	0.6919	3.524	67.5609	123.6958
Exp-1 + Exp-7 + Exp-3 + Exp-4	Grain Yield	0.5507	7378	5.1066	26.6976	0.8599	5.7514	75.0092	98.3613
Exp-1 + Exp-7 + Exp-3 + Exp-2	Grain Yield	0.5068	3857	4.9529	23.6314	0.8681	7.1277	57.7074	66.8372
Exp-1 + Exp-7 + Exp-4 + Exp-2	Grain Yield	0.5322	6498	4.1521	21.4883	0.8109	5.1046	70.7197	91.8839
Exp-1 + Exp-8 + Exp-3 + Exp-4	Grain Yield	0.5396	10604	5.0179	29.6437	0.8199	4.0099	79.931	132.6644
Exp-1 + Exp-8 + Exp-3 + Exp-2	Grain Yield	0.5657	7082	4.8655	28.7652	0.8037	3.8995	68.7707	102.9799
Exp-1 + Exp-8 + Exp-4 + Exp-2	Grain Yield	0.5283	9724	4.1138	24.9096	0.7573	3.4192	76.7098	126.7635
Exp-1 + Exp-3 + Exp-4 + Exp-2	Grain Yield	0.4593	5193	5.8284	32.921	0.8841	6.0417	73.4562	70.6952
Exp-5 + Exp-6 + Exp-7 + Exp-8	Grain Yield	0.6272	12939	3.37	16.5899	0.7012	4.0254	78.3774	165.0859
Exp-5 + Exp-6 + Exp-7 + Exp-3	Grain Yield	0.6152	8467	4.2356	19.2678	0.8068	5.9247	79.4695	106.5440
Exp-5 + Exp-6 + Exp-7 + Exp-4	Grain Yield	0.6089	11092	3.9596	19.0072	0.779	5.0317	84.7516	130.8766
Exp-5 + Exp-6 + Exp-7 + Exp-2	Grain Yield	0.6223	7540	3.4761	15.1242	0.7428	5.4244	74.3666	101.3896
Exp-5 + Exp-6 + Exp-8 + Exp-3	Grain Yield	0.6175	11693	4.1668	21.6785	0.765	4.2972	84.1102	139.0200

Exp-5 + Exp-6 + Exp-8 + Exp-4	Grain Yield	0.6102	14318	3.9115	20.916	0.7424	3.9024	88.9474	160.9715
Exp-5 + Exp-6 + Exp-8 + Exp-2	Grain Yield	0.6389	10767	3.4006	17.3985	0.6857	3.8059	79.7405	135.0255
Exp-5 + Exp-6 + Exp-3 + Exp-4	Grain Yield	0.5871	9811	4.7906	24.2132	0.8286	5.5025	90.0084	109.0009
Exp-5 + Exp-6 + Exp-3 + Exp-2	Grain Yield	0.6005	6284	4.5646	21.5064	0.8224	6.2163	83.2233	75.5077
Exp-5 + Exp-6 + Exp-4 + Exp-2	Grain Yield	0.571	8914	4.1395	20.5053	0.7832	5.0157	86.5892	102.9459
Exp-5 + Exp-7 + Exp-8 + Exp-3	Grain Yield	0.582	12076	4.1138	21.4938	0.7685	4.3823	75.3319	160.3039
Exp-5 + Exp-7 + Exp-8 + Exp-4	Grain Yield	0.5764	14702	3.8031	20.8034	0.7467	3.9825	80.9993	181.5077
Exp-5 + Exp-7 + Exp-8 + Exp-2	Grain Yield	0.5894	11150	3.3261	17.4192	0.695	3.9148	71.0496	156.9326
Exp-5 + Exp-7 + Exp-3 + Exp-4	Grain Yield	0.5742	10195	4.7045	23.9301	0.8292	5.5563	79.7502	127.8367
Exp-5 + Exp-7 + Exp-3 + Exp-2	Grain Yield	0.5535	6668	4.4917	21.2145	0.8229	6.2568	69.0874	96.5154
Exp-5 + Exp-7 + Exp-4 + Exp-2	Grain Yield	0.5665	9299	4.0025	20.3518	0.7861	5.0946	75.9493	122.4369
Exp-5 + Exp-8 + Exp-3 + Exp-4	Grain Yield	0.5686	13421	4.6007	25.89	0.7939	4.226	85.0884	157.7301
Exp-5 + Exp-8 + Exp-3 + Exp-2	Grain Yield	0.5728	9894	4.3403	23.8714	0.77	4.2259	75.578	130.9111
Exp-5 + Exp-8 + Exp-4 + Exp-2	Grain Yield	0.5611	12525	3.9067	22.3835	0.7409	3.7875	81.7074	153.2909
Exp-5 + Exp-3 + Exp-4 + Exp-2	Grain Yield	0.5254	8012	5.088	27.0405	0.8426	5.6848	78.7938	101.6831

Exp-6 + Exp-7 + Exp-8 + Exp-3	Grain Yield	0.5927	10851	4.0647	20.678	0.7707	4.2022	78.7477	137.7945
Exp-6 + Exp-7 + Exp-8 + Exp-4	Grain Yield	0.5752	13477	3.838	20.1411	0.7483	3.8014	83.8727	160.6840
Exp-6 + Exp-7 + Exp-8 + Exp-2	Grain Yield	0.6173	9922	3.1364	15.3364	0.6739	3.6618	75.4717	131.4665
Exp-6 + Exp-7 + Exp-3 + Exp-4	Grain Yield	0.5805	8972	4.7084	23.2741	0.8366	5.4975	85.1037	105.4243
Exp-6 + Exp-7 + Exp-3 + Exp-2	Grain Yield	0.5942	5443	4.2977	19.1679	0.8261	6.3192	73.7347	73.8187
Exp-6 + Exp-7 + Exp-4 + Exp-2	Grain Yield	0.5833	8074	3.9637	18.8931	0.7868	4.9612	81.0612	99.6038
Exp-6 + Exp-8 + Exp-3 + Exp-4	Grain Yield	0.5823	12198	4.6866	26.0233	0.8021	4.0496	88.1372	138.3979
Exp-6 + Exp-8 + Exp-3 + Exp-2	Grain Yield	0.6143	8668	4.3262	23.4185	0.7746	3.9793	79.6829	108.7812
Exp-6 + Exp-8 + Exp-4 + Exp-2	Grain Yield	0.579	11300	3.9744	21.8603	0.7426	3.5505	85.0468	132.8680
Exp-6 + Exp-3 + Exp-4 + Exp-2	Grain Yield	0.5483	6789	5.1506	26.8352	0.8538	5.6317	87.346	77.7254
Exp-7 + Exp-8 + Exp-3 + Exp-4	Grain Yield	0.5595	12581	4.6032	25.6889	0.8042	4.1387	79.7424	157.7705
Exp-7 + Exp-8 + Exp-3 + Exp-2	Grain Yield	0.576	9052	4.2682	23.0579	0.7788	4.1055	69.925	129.4530
Exp-7 + Exp-8 + Exp-4 + Exp-2	Grain Yield	0.5546	11684	3.8419	21.6826	0.7484	3.6628	76.5379	152.6564
Exp-7 + Exp-3 + Exp-4 + Exp-2	Grain Yield	0.5591	7173	5.0427	26.3133	0.8535	5.6996	74.7299	95.9857
Exp-8 + Exp-3 + Exp-4 + Exp-2	Grain Yield	0.5485	10399	4.9372	29.1955	0.8101	3.9397	79.6301	130.5913

Exp-1 + Exp-5 + Exp-6 + Exp-7 + Exp-8	Grain Yield	0.6227	13306	3.4421	16.9848	0.714	4.1375	80.2904	165.7234
Exp-1 + Exp-5 + Exp-6 + Exp-7 + Exp-3	Grain Yield	0.6167	8809	4.2775	19.5664	0.8131	6.0314	80.8795	108.9151
Exp-1 + Exp-5 + Exp-6 + Exp-7 + Exp-4	Grain Yield	0.6031	11447	3.9929	19.2046	0.7851	5.135	85.8856	133.2819
Exp-1 + Exp-5 + Exp-6 + Exp-7 + Exp-2	Grain Yield	0.6224	7902	3.5621	15.6257	0.7556	5.5514	76.9891	102.6379
Exp-1 + Exp-5 + Exp-6 + Exp-8 + Exp-3	Grain Yield	0.6352	12035	4.2117	21.9274	0.774	4.4217	85.4157	140.8992
Exp-1 + Exp-5 + Exp-6 + Exp-8 + Exp-4	Grain Yield	0.612	14673	3.9465	21.078	0.7505	4.0103	89.9827	163.0647
Exp-1 + Exp-5 + Exp-6 + Exp-8 + Exp-2	Grain Yield	0.6493	11129	3.4923	17.8829	0.7032	3.9489	81.9029	135.8804
Exp-1 + Exp-5 + Exp-6 + Exp-3 + Exp-4	Grain Yield	0.5958	10145	4.8012	24.2934	0.8329	5.6139	90.3836	112.2438
Exp-1 + Exp-5 + Exp-6 + Exp-3 + Exp-2	Grain Yield	0.6089	6624	4.6	21.7635	0.8289	6.3451	84.2547	78.6188
Exp-1 + Exp-5 + Exp-6 + Exp-4 + Exp-2	Grain Yield	0.5912	9266	4.1733	20.6863	0.7905	5.1455	87.6342	105.7350
Exp-1 + Exp-5 + Exp-7 + Exp-8 + Exp-3	Grain Yield	0.5875	12418	4.1558	21.7372	0.7768	4.5006	76.6325	162.0461
Exp-1 + Exp-5 + Exp-7 + Exp-8 + Exp-4	Grain Yield	0.5811	15057	3.841	20.9609	0.7542	4.0858	82.071	183.4631
Exp-1 + Exp-5 + Exp-7 + Exp-8 + Exp-2	Grain Yield	0.6035	11512	3.4138	17.8695	0.7107	4.0496	73.0696	157.5484
Exp-1 + Exp-5 + Exp-7 + Exp-3 + Exp-4	Grain Yield	0.5662	10529	4.7153	24.0184	0.8332	5.6619	80.4253	130.9165
Exp-1 + Exp-5 + Exp-7 + Exp-3 + Exp-2	Grain Yield	0.5643	7008	4.5241	21.475	0.829	6.3765	70.5256	99.3682

Exp-1 + Exp-5 + Exp-7 + Exp-4 + Exp-2	Grain Yield	0.575	9651	4.0411	20.5314	0.7929	5.2162	77.2145	124.9895
Exp-1 + Exp-5 + Exp-8 + Exp-3 + Exp-4	Grain Yield	0.5623	13755	4.6166	25.947	0.7999	4.3392	85.8001	160.3145
Exp-1 + Exp-5 + Exp-8 + Exp-3 + Exp-2	Grain Yield	0.5868	10234	4.3835	24.0828	0.7804	4.3755	76.9884	132.9291
Exp-1 + Exp-5 + Exp-8 + Exp-4 + Exp-2	Grain Yield	0.5741	12877	3.9514	22.5358	0.7507	3.9146	82.8732	155.3820
Exp-1 + Exp-5 + Exp-3 + Exp-4 + Exp-2	Grain Yield	0.526	8344	5.083	27.005	0.8468	5.8142	79.2474	105.2905
Exp-1 + Exp-6 + Exp-7 + Exp-8 + Exp-3	Grain Yield	0.6041	11195	4.116	20.9803	0.7799	4.3382	79.7191	140.4306
Exp-1 + Exp-6 + Exp-7 + Exp-8 + Exp-4	Grain Yield	0.5767	13832	3.8731	20.3142	0.7562	3.9185	84.886	162.9480
Exp-1 + Exp-6 + Exp-7 + Exp-8 + Exp-2	Grain Yield	0.6045	10286	3.2615	16.0355	0.6957	3.8208	77.175	133.2815
Exp-1 + Exp-6 + Exp-7 + Exp-3 + Exp-4	Grain Yield	0.5858	9306	4.7209	23.3829	0.8404	5.619	85.7525	108.5216
Exp-1 + Exp-6 + Exp-7 + Exp-3 + Exp-2	Grain Yield	0.5963	5785	4.3597	19.6167	0.8333	6.4583	75.0357	77.0966
Exp-1 + Exp-6 + Exp-7 + Exp-4 + Exp-2	Grain Yield	0.5789	8426	4.0069	19.154	0.7943	5.1061	82.4683	102.1726
Exp-1 + Exp-6 + Exp-8 + Exp-3 + Exp-4	Grain Yield	0.5881	12532	4.6974	26.0449	0.8079	4.1786	88.7515	141.2032
Exp-1 + Exp-6 + Exp-8 + Exp-3 + Exp-2	Grain Yield	0.6278	9010	4.3783	23.6667	0.786	4.1577	80.6447	111.7246
Exp-1 + Exp-6 + Exp-8 + Exp-4 + Exp-2	Grain Yield	0.5868	11652	4.014	22.019	0.7531	3.6982	86.1514	135.2503
Exp-1 + Exp-6 + Exp-3 + Exp-4 + Exp-2	Grain Yield	0.5305	7121	5.142	26.7674	0.8574	5.7859	87.6694	81.2256

Exp-1 + Exp-7 + Exp-8 + Exp-3 + Exp-4	Grain Yield	0.5542	12915	4.6143	25.7213	0.8095	4.2615	80.4771	160.4804
Exp-1 + Exp-7 + Exp-8 + Exp-3 + Exp-2	Grain Yield	0.5727	9394	4.3152	23.3064	0.7892	4.2719	71.0377	132.2396
Exp-1 + Exp-7 + Exp-8 + Exp-4 + Exp-2	Grain Yield	0.5553	12036	3.8859	21.8368	0.758	3.8023	77.7029	154.8977
Exp-1 + Exp-7 + Exp-3 + Exp-4 + Exp-2	Grain Yield	0.548	7505	5.0351	26.2783	0.8569	5.8428	75.6173	99.2498
Exp-1 + Exp-8 + Exp-3 + Exp-4 + Exp-2	Grain Yield	0.5487	10731	4.9362	29.087	0.8166	4.0945	80.4165	133.4428
Exp-5 + Exp-6 + Exp-7 + Exp-8 + Exp-3	Grain Yield	0.6102	14004	3.968	19.8491	0.7619	4.4825	82.9509	168.8228
Exp-5 + Exp-6 + Exp-7 + Exp-8 + Exp-4	Grain Yield	0.6088	16630	3.7846	19.5425	0.7445	4.1133	87.5388	189.9729
Exp-5 + Exp-6 + Exp-7 + Exp-8 + Exp-2	Grain Yield	0.6276	13079	3.3414	16.4179	0.6992	4.0907	79.1752	165.1906
Exp-5 + Exp-6 + Exp-7 + Exp-3 + Exp-4	Grain Yield	0.6093	12123	4.4841	21.9308	0.8169	5.4857	88.5592	136.8915
Exp-5 + Exp-6 + Exp-7 + Exp-3 + Exp-2	Grain Yield	0.6229	8596	4.2	19.1129	0.8048	6.0006	80.3269	107.0127
Exp-5 + Exp-6 + Exp-7 + Exp-4 + Exp-2	Grain Yield	0.5987	11227	3.931	18.8527	0.7771	5.0975	85.3068	131.6073
Exp-5 + Exp-6 + Exp-8 + Exp-3 + Exp-4	Grain Yield	0.604	15349	4.4236	23.7347	0.787	4.3371	92.3435	166.2164
Exp-5 + Exp-6 + Exp-8 + Exp-3 + Exp-2	Grain Yield	0.6201	11822	4.1217	21.415	0.7629	4.3702	84.8236	139.3716
Exp-5 + Exp-6 + Exp-8 + Exp-4 + Exp-2	Grain Yield	0.6066	14453	3.8759	20.681	0.7404	3.964	89.4343	161.6047
Exp-5 + Exp-6 + Exp-3 + Exp-4 + Exp-2	Grain Yield	0.5922	9940	4.7478	23.9818	0.8269	5.5737	90.4303	109.9189

Exp-5 + Exp-7 + Exp-8 + Exp-3 + Exp-4	Grain Yield	0.5833	15732	4.3564	23.5422	0.7891	4.4015	84.4519	186.2836
Exp-5 + Exp-7 + Exp-8 + Exp-3 + Exp-2	Grain Yield	0.5934	12205	4.0714	21.2423	0.7664	4.4521	76.0037	160.5843
Exp-5 + Exp-7 + Exp-8 + Exp-4 + Exp-2	Grain Yield	0.5782	14837	3.7768	20.5774	0.7446	4.0418	81.4888	182.0741
Exp-5 + Exp-7 + Exp-3 + Exp-4 + Exp-2	Grain Yield	0.5778	10324	4.6646	23.7091	0.8275	5.6242	80.2414	128.6618
Exp-5 + Exp-8 + Exp-3 + Exp-4 + Exp-2	Grain Yield	0.5662	13550	4.5564	25.5985	0.7921	4.2904	85.5345	158.4156
Exp-6 + Exp-7 + Exp-8 + Exp-3 + Exp-4	Grain Yield	0.5784	14509	4.3898	23.2779	0.7943	4.2678	87.1008	166.5771
Exp-6 + Exp-7 + Exp-8 + Exp-3 + Exp-2	Grain Yield	0.6046	10980	4.0029	20.3195	0.7667	4.2819	79.3265	138.4153
Exp-6 + Exp-7 + Exp-8 + Exp-4 + Exp-2	Grain Yield	0.5818	13612	3.7889	19.8273	0.7445	3.8678	84.3475	161.3800
Exp-6 + Exp-7 + Exp-3 + Exp-4 + Exp-2	Grain Yield	0.5858	9101	4.6571	22.9974	0.8339	5.5753	85.6393	106.2713
Exp-6 + Exp-8 + Exp-3 + Exp-4 + Exp-2	Grain Yield	0.5949	12327	4.6235	25.6206	0.799	4.1222	88.5755	139.1694
Exp-7 + Exp-8 + Exp-3 + Exp-4 + Exp-2	Grain Yield	0.5743	12710	4.5449	25.3067	0.8012	4.2082	80.196	158.4867
Exp-1 + Exp-5 + Exp-6 + Exp-7 + Exp-8 + Exp-3	Grain Yield	0.6151	14346	4.0109	20.103	0.7695	4.5826	84.0913	170.6003
Exp-1 + Exp-5 + Exp-6 + Exp-7 + Exp-8 + Exp-4	Grain Yield	0.6029	16985	3.8175	19.7109	0.7512	4.2021	88.4652	191.9964
Exp-1 + Exp-5 + Exp-6 + Exp-7 + Exp-8 + Exp-2	Grain Yield	0.6314	13441	3.4144	16.8152	0.712	4.2014	80.9499	166.0410
Exp-1 + Exp-5 + Exp-6 + Exp-7 + Exp-3 + Exp-4	Grain Yield	0.6046	12457	4.5025	22.0652	0.8209	5.5768	89.0934	139.8196

Exp-1 + Exp-5 + Exp-6 + Exp-7 + Exp-3 + Exp-2	Grain Yield	0.6199	8936	4.243	19.4103	0.8112	6.1043	81.57	109.5501
Exp-1 + Exp-5 + Exp-6 + Exp-7 + Exp-4 + Exp-2	Grain Yield	0.6101	11579	3.9659	19.0551	0.7834	5.1988	86.3544	134.0870
Exp-1 + Exp-5 + Exp-6 + Exp-8 + Exp-3 + Exp-4	Grain Yield	0.6015	15683	4.4439	23.8377	0.7925	4.4341	92.919	168.7814
Exp-1 + Exp-5 + Exp-6 + Exp-8 + Exp-3 + Exp-2	Grain Yield	0.6329	12162	4.1682	21.6697	0.7719	4.4921	86.0147	141.3944
Exp-1 + Exp-5 + Exp-6 + Exp-8 + Exp-4 + Exp-2	Grain Yield	0.614	14805	3.913	20.8538	0.7486	4.0703	90.409	163.7558
Exp-1 + Exp-5 + Exp-6 + Exp-3 + Exp-4 + Exp-2	Grain Yield	0.5913	10272	4.7596	24.0625	0.8312	5.6825	90.7322	113.2123
Exp-1 + Exp-5 + Exp-7 + Exp-8 + Exp-3 + Exp-4	Grain Yield	0.5822	16066	4.3767	23.6467	0.7942	4.4948	85.1165	188.7531
Exp-1 + Exp-5 + Exp-7 + Exp-8 + Exp-3 + Exp-2	Grain Yield	0.5862	12545	4.1152	21.4908	0.7748	4.568	77.2119	162.4750
Exp-1 + Exp-5 + Exp-7 + Exp-8 + Exp-4 + Exp-2	Grain Yield	0.5778	15189	3.816	20.7449	0.7524	4.1436	82.5075	184.0924
Exp-1 + Exp-5 + Exp-7 + Exp-3 + Exp-4 + Exp-2	Grain Yield	0.5794	10656	4.6766	23.7974	0.8316	5.7274	80.8516	131.7970
Exp-1 + Exp-5 + Exp-8 + Exp-3 + Exp-4 + Exp-2	Grain Yield	0.5626	13882	4.5737	25.6596	0.7981	4.4017	86.1952	161.0531
Exp-1 + Exp-6 + Exp-7 + Exp-8 + Exp-3 + Exp-4	Grain Yield	0.5807	14843	4.409	23.3809	0.7994	4.3718	87.6767	169.2924
Exp-1 + Exp-6 + Exp-7 + Exp-8 + Exp-3 + Exp-2	Grain Yield	0.6144	11322	4.0579	20.6385	0.7762	4.4147	80.2275	141.1237
Exp-1 + Exp-6 + Exp-7 + Exp-8 + Exp-4 + Exp-2	Grain Yield	0.5844	13964	3.828	20.0217	0.7528	3.9829	85.3033	163.6982
Exp-1 + Exp-6 + Exp-7 + Exp-3 + Exp-4 + Exp-2	Grain Yield	0.5832	9433	4.6715	23.11	0.8379	5.6936	86.2037	109.4269

Exp-1 + Exp-6 + Exp-8 + Exp-3 + Exp-4 + Exp-2	Grain Yield	0.5905	12659	4.6374	25.6566	0.805	4.2487	89.1327	142.0242
Exp-1 + Exp-7 + Exp-8 + Exp-3 + Exp-4 + Exp-2	Grain Yield	0.5679	13042	4.5589	25.3516	0.8067	4.3286	80.8817	161.2478
Exp-5 + Exp-6 + Exp-7 + Exp-8 + Exp-3 + Exp-4	Grain Yield	0.6055	17660	4.2331	21.993	0.7829	4.479	90.5918	194.9404
Exp-5 + Exp-6 + Exp-7 + Exp-8 + Exp-3 + Exp-2	Grain Yield	0.6194	14133	3.9347	19.6618	0.7601	4.5419	83.5441	169.1681
Exp-5 + Exp-6 + Exp-7 + Exp-8 + Exp-4 + Exp-2	Grain Yield	0.6029	16765	3.757	19.3661	0.7427	4.1647	87.9656	190.5859
Exp-5 + Exp-6 + Exp-7 + Exp-3 + Exp-4 + Exp-2	Grain Yield	0.6134	12252	4.4528	21.7678	0.8154	5.5436	88.9693	137.7104
Exp-5 + Exp-6 + Exp-8 + Exp-3 + Exp-4 + Exp-2	Grain Yield	0.6123	15478	4.3875	23.5118	0.7854	4.3926	92.7213	166.9304
Exp-5 + Exp-7 + Exp-8 + Exp-3 + Exp-4 + Exp-2	Grain Yield	0.5875	15861	4.3234	23.3273	0.7875	4.4551	84.8436	186.9440
Exp-6 + Exp-7 + Exp-8 + Exp-3 + Exp-4 + Exp-2	Grain Yield	0.5868	14638	4.3445	22.9984	0.7917	4.327	87.4773	167.3348
Exp-1 + Exp-5 + Exp-6 + Exp-7 + Exp-8 + Exp-3 + Exp-4	Grain Yield	0.6081	17994	4.2552	22.1197	0.7877	4.5608	91.155	197.4000
Exp-1 + Exp-5 + Exp-6 + Exp-7 + Exp-8 + Exp-3 + Exp-2	Grain Yield	0.6255	14473	3.9785	19.9178	0.7677	4.6403	84.6014	171.0728
Exp-1 + Exp-5 + Exp-6 + Exp-7 + Exp-8 + Exp-4 + Exp-2	Grain Yield	0.5993	17117	3.7913	19.5412	0.7495	4.2525	88.846	192.6592

Exp-1 + Exp-5 + Exp-6 + Exp-7 + Exp-3 + Exp-4 + Exp-2	Grain Yield	0.6065	12584	4.4717	21.9003	0.8194	5.6332	89.447	140.6867
Exp-1 + Exp-5 + Exp-6 + Exp-8 + Exp-3 + Exp-4 + Exp-2	Grain Yield	0.6153	15810	4.4085	23.6162	0.7909	4.4882	93.2519	169.5408
Exp-1 + Exp-5 + Exp-7 + Exp-8 + Exp-3 + Exp-4 + Exp-2	Grain Yield	0.5919	16193	4.3443	23.4328	0.7926	4.5471	85.4688	189.4609
Exp-1 + Exp-6 + Exp-7 + Exp-8 + Exp-3 + Exp-4 + Exp-2	Grain Yield	0.5892	14970	4.3652	23.1072	0.797	4.4294	88.0108	170.0928
Exp-5 + Exp-6 + Exp-7 + Exp-8 + Exp-3 + Exp-4 + Exp-2	Grain Yield	0.6107	17789	4.2048	21.8225	0.7814	4.5262	90.9334	195.6267
Exp-1 + Exp-5 + Exp-6 + Exp-7 + Exp-8 + Exp-3 + Exp-4 + Exp-2	Grain Yield	0.6094	18121	4.2273	21.9488	0.7862	4.6071	91.4613	198.1275
Exp-1	Plant Height	0.4859	369	10.2435	80.9622	0.9861	8.0216	36.845	10.0149
Exp-5	Plant Height	0.6451	3158	5.6279	26.748	0.9294	5.5906	68.8316	45.8801
Exp-6	Plant Height	0.548	1710	3.9609	16.5103	0.7608	2.5175	74.5207	22.9466
Exp-7	Plant Height	0.584	2276	4.5961	21.1575	0.8866	3.7562	43.5122	52.3072

Exp-8	Plant Height	0.5422	3384	5.4223	34.332	0.8194	1.8287	51.0921	66.2333
Exp-3	Plant Height	0.5392	464	3.2281	8.5396	0.9274	19.591	71.3502	6.5031
Exp-1 + Exp-5	Plant Height	0.6643	3525	6.2941	32.7167	0.9456	5.85	68.6001	51.3848
Exp-1 + Exp-6	Plant Height	0.6142	2079	5.9348	30.8714	0.9073	3.495	75.728	27.4535
Exp-1 + Exp-7	Plant Height	0.6582	2645	5.7882	30.6961	0.9314	4.351	48.5736	54.4534
Exp-1 + Exp-8	Plant Height	0.6123	3753	6.4738	42.2566	0.9004	2.4399	54.6402	68.6857
Exp-1 + Exp-3	Plant Height	0.5451	832	7.168	39.8283	0.9793	14.5036	59.1634	14.0627
Exp-5 + Exp-6	Plant Height	0.6342	4868	5.2104	23.8205	0.9027	4.5167	72.9412	66.7387
Exp-5 + Exp-7	Plant Height	0.6627	5433	5.2101	24.527	0.9151	4.83	60.3942	89.9590
Exp-5 + Exp-8	Plant Height	0.6231	6542	5.6568	30.6812	0.9046	3.6503	61.6514	106.1128
Exp-5 + Exp-3	Plant Height	0.6766	3621	5.355	24.0242	0.9365	7.3929	71.8379	50.4051
Exp-6 + Exp-7	Plant Height	0.6167	3986	4.4455	19.4426	0.8508	3.231	63.2422	63.0275
Exp-6 + Exp-8	Plant Height	0.5969	5094	5.0724	27.3806	0.8047	2.0619	66.4673	76.6392
Exp-6 + Exp-3	Plant Height	0.63	2174	3.6735	13.8339	0.8664	6.1694	82.9487	26.2090
Exp-7 + Exp-8	Plant Height	0.6007	5659	5.2252	29.1371	0.8697	2.6106	56.4566	100.2363

Exp-7 + Exp-3	Plant Height	0.6156	2740	4.3334	17.8842	0.9064	6.4386	52.1329	52.5580
Exp-8 + Exp-3	Plant Height	0.5854	3848	5.3796	29.6933	0.898	3.9803	57.1857	67.2895
Exp-1 + Exp-5 + Exp-6	Plant Height	0.6638	5235	5.7607	28.3291	0.923	4.7668	77.0445	67.9477
Exp-1 + Exp-5 + Exp-7	Plant Height	0.7028	5800	5.6904	28.496	0.9302	5.0357	63.9995	90.6257
Exp-1 + Exp-5 + Exp-8	Plant Height	0.6586	6909	6.0749	34.2206	0.9209	3.886	65.163	106.0264
Exp-1 + Exp-5 + Exp-3	Plant Height	0.6985	3987	6.0014	29.5689	0.9494	7.4585	71.7879	55.5386
Exp-1 + Exp-6 + Exp-7	Plant Height	0.6664	4355	5.3089	25.8858	0.8993	3.6376	67.0935	64.9094
Exp-1 + Exp-6 + Exp-8	Plant Height	0.636	5463	5.8731	33.6056	0.8734	2.466	69.4662	78.6426
Exp-1 + Exp-6 + Exp-3	Plant Height	0.6413	2542	5.4068	25.8654	0.931	6.4434	81.4197	31.2209
Exp-1 + Exp-7 + Exp-8	Plant Height	0.668	6028	5.8042	33.7384	0.9018	2.9428	59.4224	101.4432
Exp-1 + Exp-7 + Exp-3	Plant Height	0.6388	3108	5.4788	26.5927	0.939	6.6304	55.2141	56.2900
Exp-1 + Exp-8 + Exp-3	Plant Height	0.6169	4216	6.2025	36.651	0.9269	4.337	59.4729	70.8894
Exp-5 + Exp-6 + Exp-7	Plant Height	0.6795	7143	5.0244	22.9757	0.8977	4.28	71.4702	99.9438
Exp-5 + Exp-6 + Exp-8	Plant Height	0.6435	8252	5.4034	27.7314	0.8876	3.4176	72.8509	113.2725
Exp-5 + Exp-6 + Exp-3	Plant Height	0.6771	5331	5.0173	22.1109	0.9141	5.8353	78.5057	67.9059

Exp-5 + Exp-7 + Exp-8	Plant Height	0.6623	8816	5.3836	28.1281	0.901	3.6831	64.9961	135.6389
Exp-5 + Exp-7 + Exp-3	Plant Height	0.66	5896	5.0796	22.899	0.9219	5.9973	65.1284	90.5289
Exp-5 + Exp-8 + Exp-3	Plant Height	0.6282	7005	5.5151	28.4594	0.916	4.7118	66.1554	105.8870
Exp-6 + Exp-7 + Exp-8	Plant Height	0.6431	7369	5.0173	25.8272	0.8498	2.5911	67.8378	108.6268
Exp-6 + Exp-7 + Exp-3	Plant Height	0.6469	4450	4.2267	17.5089	0.8775	4.9412	69.3762	64.1430
Exp-6 + Exp-8 + Exp-3	Plant Height	0.6031	5558	4.9215	24.9316	0.8675	3.532	71.337	77.9119
Exp-7 + Exp-8 + Exp-3	Plant Height	0.6234	6123	5.0799	26.1908	0.8925	3.9024	60.822	100.6708
Exp-1 + Exp-5 + Exp-6 + Exp-7	Plant Height	0.6911	7510	5.4384	26.2573	0.9143	4.4658	74.5194	100.7791
Exp-1 + Exp-5 + Exp-6 + Exp-8	Plant Height	0.6643	8619	5.7763	30.8029	0.9055	3.6165	75.8159	113.6833
Exp-1 + Exp-5 + Exp-6 + Exp-3	Plant Height	0.6818	5697	5.5558	26.3804	0.9301	5.9813	81.0187	70.3171
Exp-1 + Exp-5 + Exp-7 + Exp-8	Plant Height	0.6934	9183	5.7201	30.8938	0.9146	3.8591	67.6234	135.7962
Exp-1 + Exp-5 + Exp-7 + Exp-3	Plant Height	0.6912	6262	5.5481	26.6921	0.9346	6.1205	67.7278	92.4583
Exp-1 + Exp-5 + Exp-8 + Exp-3	Plant Height	0.6488	7371	5.9228	31.9213	0.9286	4.8805	68.9062	106.9715
Exp-1 + Exp-6 + Exp-7 + Exp-8	Plant Height	0.6762	7738	5.5195	29.764	0.883	2.851	70.3163	110.0456
Exp-1 + Exp-6 + Exp-7 + Exp-3	Plant Height	0.6569	4818	5.0735	23.5728	0.9128	5.1793	71.7484	67.1513

Exp-1 + Exp-6 + Exp-8 + Exp-3	Plant Height	0.6365	5926	5.6285	30.4851	0.9018	3.8136	73.3604	80.7793
Exp-1 + Exp-7 + Exp-8 + Exp-3	Plant Height	0.6464	6491	5.6435	30.7516	0.9149	4.1382	63.0286	102.9850
Exp-5 + Exp-6 + Exp-7 + Exp-8	Plant Height	0.6814	10526	5.2323	26.2777	0.8882	3.4955	73.5894	143.0369
Exp-5 + Exp-6 + Exp-7 + Exp-3	Plant Height	0.6861	7606	4.9022	21.7589	0.9063	5.2189	75.2788	101.0377
Exp-5 + Exp-6 + Exp-8 + Exp-3	Plant Height	0.6545	8715	5.2669	26.1345	0.9004	4.2835	76.5284	113.8793
Exp-5 + Exp-7 + Exp-8 + Exp-3	Plant Height	0.6622	9279	5.2899	26.5729	0.9093	4.483	68.2425	135.9710
Exp-6 + Exp-7 + Exp-8 + Exp-3	Plant Height	0.6573	7833	4.8591	23.8757	0.8736	3.6025	71.4422	109.6411
Exp-1 + Exp-5 + Exp-6 + Exp-7 + Exp-8	Plant Height	0.699	10893	5.5373	28.7363	0.9026	3.6502	75.8954	143.5265
Exp-1 + Exp-5 + Exp-6 + Exp-7 + Exp-3	Plant Height	0.6899	7972	5.3107	24.9231	0.9204	5.3515	77.5841	102.7530
Exp-1 + Exp-5 + Exp-6 + Exp-8 + Exp-3	Plant Height	0.6648	9081	5.6353	29.127	0.9145	4.4377	78.8813	115.1223
Exp-1 + Exp-5 + Exp-7 + Exp-8 + Exp-3	Plant Height	0.6819	9645	5.6213	29.2932	0.9207	4.6206	70.4117	136.9801
Exp-1 + Exp-6 + Exp-7 + Exp-8 + Exp-3	Plant Height	0.6633	8201	5.3546	27.7085	0.8978	3.8027	73.3023	111.8792
Exp-5 + Exp-6 + Exp-7 + Exp-8 + Exp-3	Plant Height	0.6762	10989	5.1331	25.0582	0.8973	4.179	76.3665	143.8982
Exp-1 + Exp-5 + Exp-6 + Exp-7 + Exp-8 + Exp-3	Plant Height	0.6792	11355	5.4363	27.4726	0.9096	4.3057	78.2791	145.0579

Supplementary Table 13: Training population size and its mean expected linkage disequilibrium (LD). The mean expected LD was calculated over 50 random samples drawn at each population size.

Training population size	Mean expected LD
500	0.00561
1000	0.00464
2500	0.00405
5000	0.00385

Supplementary figures

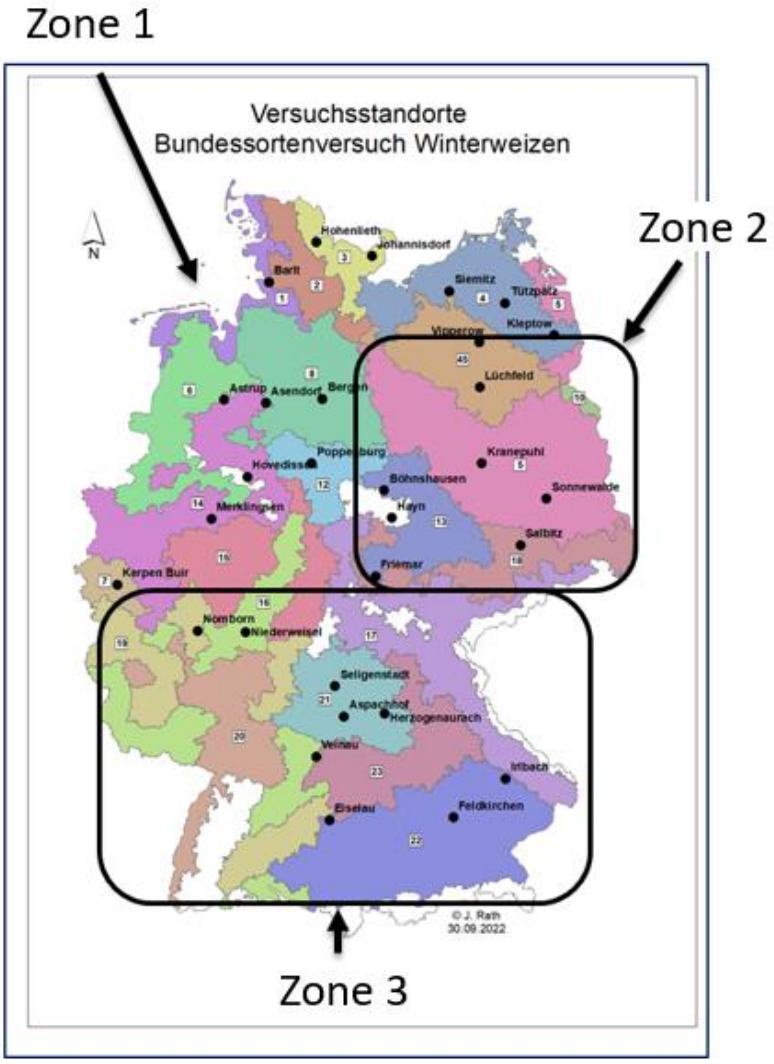

Supplementary Fig. 1: A geographical map of Germany marked with the locations used in Exp-PRT and the zones they fall under.

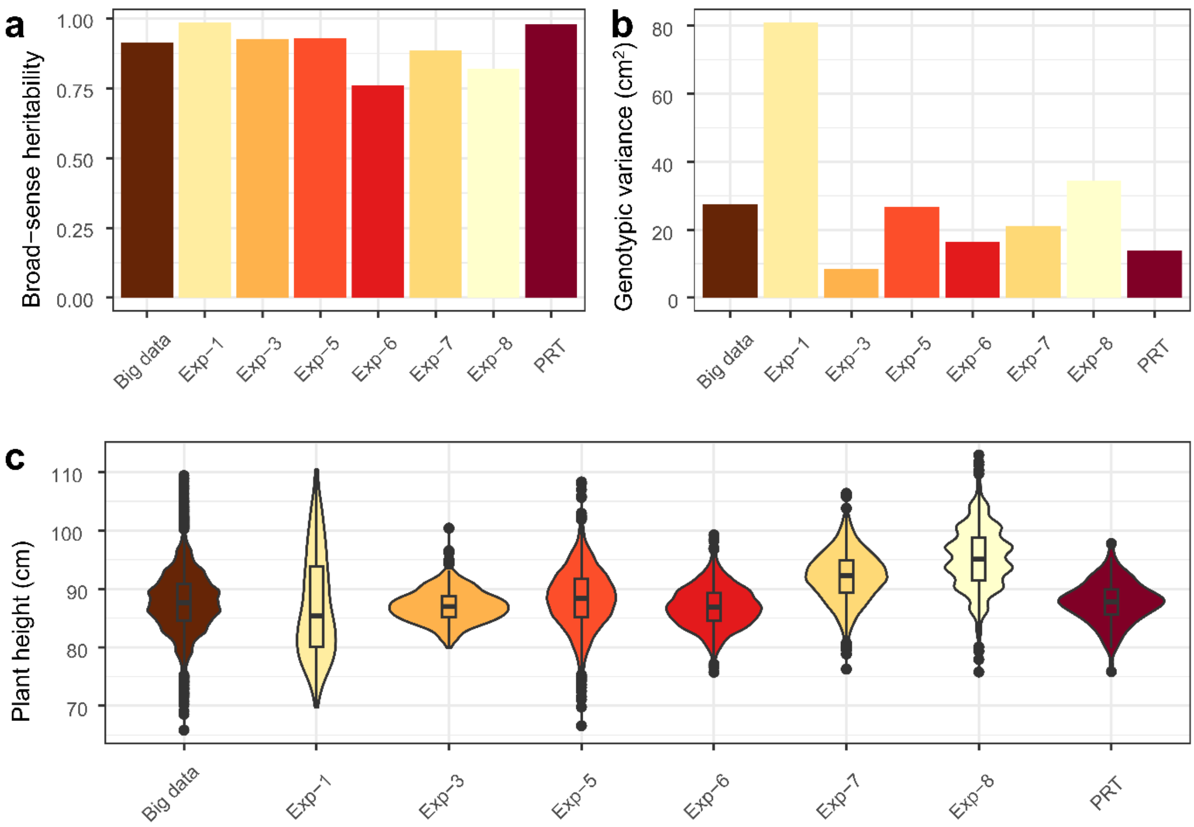

Supplementary Fig. 2: Within and across experimental series (a) Broad-sense heritabilities, (b) Variance due to genotypes and (c) Distribution of best linear unbiased predictions (BLUPs) of plant height (cm).

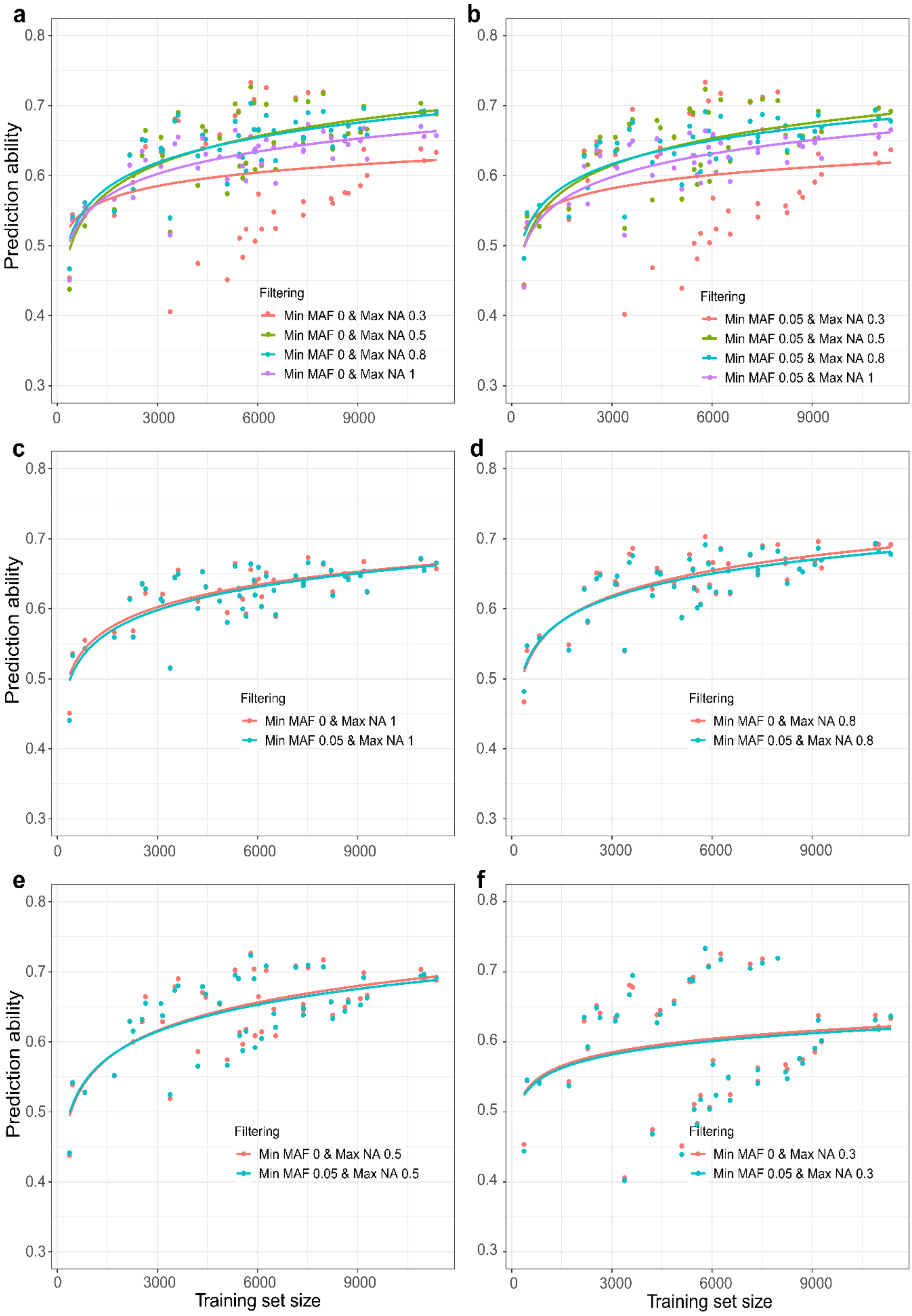

Supplementary Fig. 3: For plant height, a-b Prediction ability vs Training set size at different NA thresholds and (a) no minor allele filtering (Min MAF 0), (b) minor allele filtering at 5% (Min MAF 0.05). c-f Prediction ability vs Training set size at different minor allele frequnecy thresholds and missing values allowed per marker (c) up to 100%, (d) up to 80%, (e) up to

*50%, and (f) up to 30%. The coloured smoothing lines indicate respective minor allele frequency and missing value filtering thresholds employed and drawn using the formula y ~ a + b * log(x).*
